# A whole-genome shotgun approach for assembling and anchoring the hexaploid bread wheat genome

**DOI:** 10.1186/s13059-015-0582-8

**Published:** 2015-01-31

**Authors:** Jarrod A Chapman, Martin Mascher, Aydın Buluç, Kerrie Barry, Evangelos Georganas, Adam Session, Veronika Strnadova, Jerry Jenkins, Sunish Sehgal, Leonid Oliker, Jeremy Schmutz, Katherine A Yelick, Uwe Scholz, Robbie Waugh, Jesse A Poland, Gary J Muehlbauer, Nils Stein, Daniel S Rokhsar

**Affiliations:** Department of Energy Joint Genome Institute, 2800 Mitchell Drive, Walnut Creek, CA 94598 USA; Leibniz Institute of Plant Genetics and Crop Plant Research (IPK) Gatersleben, 06466 Stadt Seeland, Germany; Computational Research Division and National Energy Research Supercomputing Center (NERSC), Lawrence Berkeley National Laboratory, Berkeley, CA 94720 USA; Department of Electrical Engineering and Computer Science, Computer Science Division, University of California, Berkeley, CA 94720 USA; Department of Molecular and Cell Biology, University of California, Berkeley, CA 94720 USA; Department of Computer Science, University of California, Santa Barbara, CA 93106 USA; HudsonAlpha Institute of Biotechnology, Huntsville, AL 35806 USA; Department of Plant Pathology, Kansas State University, Manhattan, KS 65506 USA; Division of Plant Sciences, University of Dundee & The James Hutton Institute, Invergowrie, Dundee DD2 5DA UK; Departments of Agronomy and Plant Genetics, and Plant Biology, University of Minnesota, St Paul, MN 55108 USA; Present address: Department of Plant Science, South Dakota State University, Brookings, SD 57007 USA

## Abstract

Polyploid species have long been thought to be recalcitrant to whole-genome assembly. By combining high-throughput sequencing, recent developments in parallel computing, and genetic mapping, we derive, *de novo*, a sequence assembly representing 9.1 Gbp of the highly repetitive 16 Gbp genome of hexaploid wheat, *Triticum aestivum*, and assign 7.1 Gb of this assembly to chromosomal locations. The genome representation and accuracy of our assembly is comparable or even exceeds that of a chromosome-by-chromosome shotgun assembly. Our assembly and mapping strategy uses only short read sequencing technology and is applicable to any species where it is possible to construct a mapping population.

**Electronic supplementary material:**

The online version of this article (doi:10.1186/s13059-015-0582-8) contains supplementary material, which is available to authorized users.

## Background

The feasibility of whole-genome shotgun (WGS) assembly of large and complex eukaryotic genomes was once a much-debated question [1,2]. The advent of next-generation sequencing and the comparative ease and speed with which WGS assemblies can be constructed for mammalian and many other genomes allowed sequencing projects to move beyond these concerns, accepting high quality draft genomes with nearly complete gene spaces. Some genomes, however, are larger and more complex than the typical mammalian genome, including those of salamanders (>20 gigabases (Gbp)) [3], hexaploid wheat (16 Gbp) [4,5], and conifers (20 Gbp) [6]. To mitigate some of the computational challenges of genome assembly from short next-generation sequencing reads for these more complex genomes, various ‘divide and conquer’ strategies have been developed. These strategies include chromosome sorting and capture [5], large-insert-clone pooling [6,7], and large-clone tiling paths [5,8]. While each approach reduces the sequence assembly problem to a set of smaller, more tractable problems, they require substantial resource development in advance of sequencing.

Many of the arguments ‘against a whole-genome shotgun’ [2] remain valid today. WGS assemblies are often rough drafts consisting of numerous, small contigs with gaps of unknown size between them. Abundant transposable elements that often form nested structures are prone to collapse in WGS assembly, resulting in an underrepresentation and mis-assembly of repetitive sequences in the final assembly [9]. The experiences derived from sequencing large and highly repetitive plant genomes have made it clear that while WGS assemblies are typically able to deliver a rough draft of the non-repetitive portion of a genome, true reference sequences with high contiguity and near-complete genome representation are only accessible following the paradigm of clone-by-clone-sequencing [10].

Despite their shortcomings, WGS approaches for large genomes [11] have important advantages that include (1) simplicity of library preparation and (2) uniformity of coverage. However, for very large (>10 Gbp), complex or polyploid genomes substantial computational resources may be required simply to manage the volume of data, and to address the challenge of resolving near-identical genomic sequences that are longer than the scale set by read length and pairing information. While the human WGS assembly [12] and other chromosome-scale mammalian assemblies (for example, mouse [13]) are computational *tours de force*, they ultimately rely on non-sequence data such as physical maps to assemble the chromosomes. The largest WGS assemblies that have been attempted to date (Norway spruce [6], white spruce [14] and loblolly pine [15], all approximately 20 Gbp) remain highly fragmented and are not yet organized into chromosomes. Importantly, whole genome assemblies of polyploid genomes have not yet been attempted. Instead, artificial diploids in the case of autopolyploids such as potato [16] or the progenitor species of allopolyploids such as wheat [17,18] and rapeseed [19] have been sequenced.

Hexaploid bread wheat (*Triticum aestivum* L., 1C = 16 Gbp, 2n = 6x = 42) is one of the most important agricultural crops, along with rice and maize. It is widely believed, however, that the hexaploid wheat genome is recalcitrant to WGS assembly and genome-wide physical mapping due to a high repeat content and potential difficulties in separating homeologous loci in the different subgenomes, which are not problems with the diploid rice [20] and maize [21] genomes. An early attempt at a WGS assembly resulted in a highly fragmented and genetically unanchored assembly [4]. Therefore, it was considered necessary to isolate individual chromosomes by flow-cytometry prior to sequencing and assembly [22]. So far, the map-based sequence of a single chromosome has been completed [23] and shotgun assemblies of the remaining 40 chromosome arms have been published [5].

Here, we describe an integrated approach to WGS assembly and genome-wide genetic mapping in hexaploid wheat. We shotgun-sequenced two unrelated individuals and a population of their recombinant progeny to varying depths, and constructed an ultra-dense genetic map. By computationally integrating the WGS assemblies and the sequence-based genetic map, we produced linked assemblies that span entire chromosomes, albeit including only the accessible non-repetitive portion of the genome. We achieved short-range contiguity (half the assembly in contigs longer than 7 to 8 kilobases) and physical linkage (half the assembly in scaffolds longer than 20 to 25 kilobases) using large-scale WGS assembly. Longer-range linkage and ordering at the chromosome scale (hundreds of megabases) is achieved through a *de novo* ultra-dense genetic linkage map based on >10 million single nucleotide polymorphism (SNP) markers. This linkage map also provides internal validation of assembly correctness. We demonstrate that this approach can be used to assemble previously intractable genomes on the scale of the large and repetitive hexaploid bread wheat genome. At the same time, we expand methods similar to those applied in diploid species such as barley [24], horseshoe crab [25] or *Caenorhabditis elegans* [26]*.*

## Results

### Whole-genome shotgun assembly

We generated a total of approximately 175-fold coverage (approximately 3 terabases) Illumina WGS sequence from two (hexaploid) bread wheat lines, ‘Synthetic W7984’ (30-fold coverage) and ‘Opata M85’ (15-fold), and a set of 90 doubled haploid (DH) lines derived from W7984/Opata F_1_ hybrids; the ‘SynOpDH’ population [27] (Tables S1, S2, and S3 in Additional file 1). Each DH line was sequenced to an average coverage of 1.4×. An existing genotyping-by-sequencing map of the SynOpDH population comprising 20,000 SNP markers provides an independent resource to validate our results [28]. We targeted W7984 for *de novo* assembly, and therefore produced more data and library types (30× coverage in paired-end and mate-pairs ranging from 250 bp to 4.5 kbp in size) for this genotype. Datasets are described in more detail in the Materials and methods section.

We assembled the 30× shotgun sequence for W7984 using an enhanced version of ‘meraculous’ [29] adapted for high performance computing (the name is a pun on the use of k-mers - contiguous nucleotide sequences of length k - to accomplish the assembly). Meraculous is a hybrid de Bruijn-graph/layout-based assembler that implements the following stages: (1) counting of k-mers, rejecting k-mers that arise from rare sequencing errors; (2) construction of a distributed mer-graph; (3) efficient traversal of the unique paths in this graph, which represent uncontested assembled segments in the genome (UUtigs); (4) organization of these paths into longer units by threading reads through these UUtigs and utilizing paired-end and mate-pair constraints; and (5) filling of residual gaps using pairing constraints. Meraculous is parallelized, can be used on a cluster or, in a new distributed implementation, on high performance systems, allowing efficient assembly of essentially arbitrarily large datasets. Based on available sequence depth we selected a basic word size k = 51 that provides sufficient k-mer depth and allows approximately 45% of the genome to be uniquely assembled (Figure S1 in Additional file 1). A small amount of prokaryotic and organellar contamination (26.8 Mbp in 17,054 scaffolds) was identified and removed.

**Figure 1:**
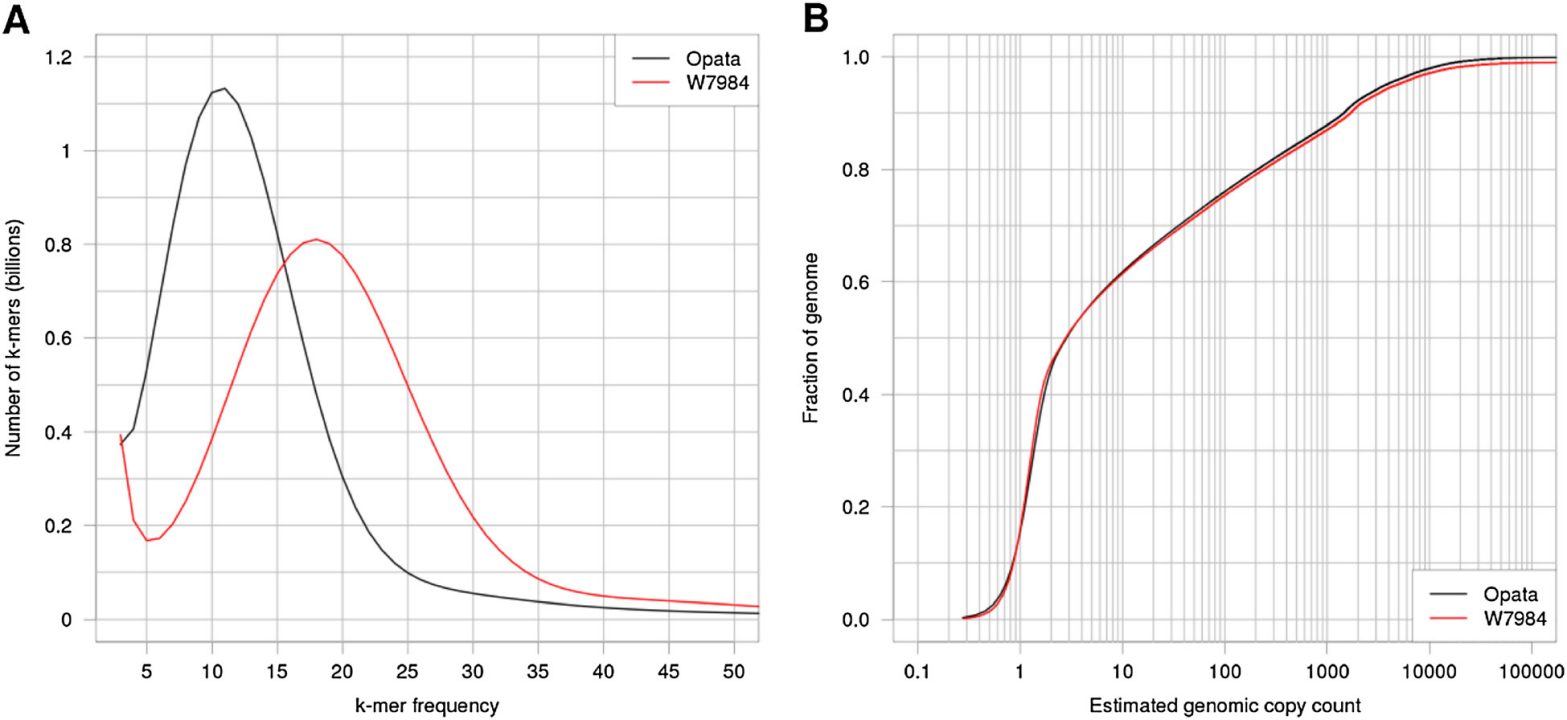
**51-mer depth distribution for homozygous parental lines. (A)** 51-mer frequency distribution for W7984 (red), compared with Opata (black). W7984 was sequenced more deeply to enable *de novo* WGS assembly. Uptick at low depth (below 51-mer frequency of approximately 5) corresponds to sequencing error. Peak frequency (approximately 18 for W7984, approximately 11 for Opata) represents the typical number of 51-mers covering nucleotides in the non-repetitive regions of the genome. **(B)** Cumulative frequency distribution for W7984 and Opata as a function of estimated genomic copy count (51-mer frequency divided by peak 51-mer frequency from panel (A)). Note logarithmic scale on the horizontal axis. The two curves lie on top of each other, as expected for two accessions from the same species. Approximately 45% of the hexaploid wheat genome is found in regions that are single copy as measured by 51-mers (estimated genomic copy count ≤2), and the remainder is typically at high 51-mer copy number (approximately 40% of the genome is found in 10 or more copies). The distribution rises smoothly through estimated genome copy counts of two and three, indicating the three subgenomes of hexaploid wheat are largely differentiated at the scale of a 51-mer.

The total estimated genome size of W7984 is 16 Gbp, consistent with prior measurements/estimates for *T. aestivum* [30]. We produced approximately 30× total sequence coverage in fragment libraries, which corresponds to approximately 18× coverage in 51-mers (Figure 1A). The very low-depth uptick (51-mer frequency below approximately 5 counts) represents sequencing errors that are easily distinguished from the error-free portion of the distribution without error correction [29].

Figure 1B shows the cumulative distribution of genome coverage as a function of relative k-mer depth, excluding the low-depth error peak. Shown on a logarithmic depth scale, it is evident that (1) the wheat genome comprises approximately 6 Gbp of 51-mer unique sequence that is accessible to de Bruijn style assembly (based on the position of the knee in this cumulative plot, approximately 6.0 Gbp is found at estimated copy number <1.5); (2) there is no clear genomic feature at double or triple copy, indicating that the A, B, and D subgenomes of hexaploid wheat are largely differentiated at k = 51; and (3) a large fraction of the genome is associated with much higher copy repeats (note logarithmic scale), which will require longer reads, or physical or optical mapping [31] approaches to assemble. For example, we estimate that approximately 4 Gbp of the genome is found at >100× copy number on a 51-bp scale. If the k-mer size is increased to 81, the unique fraction of the genome at this k-mer scale would increase to approximately 10 Gbp (Figure S1 in Additional file 1), suggesting that additional sequence depth at current read lengths would increase the assembled sequence.

We emphasize that the cumulative k-mer depth distribution shown in Figure 1B is only a rough guide to the outcome of an assembly, since it does not capture the distribution of repetitive sequences across the genome. For example, multi-copy k-mers that are embedded in otherwise k-mer unique sequence can generally be assembled using paired-end information, since their non-repetitive contexts can be established by flanking sequence. In a specific case of interest for wheat, any exons that are identical between homeologs can be assembled into their appropriate loci based on the more divergent surrounding intronic and intergenic sequence. Conversely, some single-copy k-mers, if embedded in otherwise highly repetitive surroundings, may only be assembled into contigs not much longer than a k-mer, and will be absent from the assembly if only substantial contigs are retained. So the estimated unique sequence derived from the knee in Figure 1B is only a rough guide.

The ‘meraculous’ WGS assembly of W7984 spans a total contig length of 7.883 Gbp and a total scaffold length of 9.117 Gbp (Table S4 in Additional file 1). (As noted above, the contig length is somewhat longer than the rough estimate of 6 Gb of unique sequence based on Figure 1B.) The difference between scaffold and contig length corresponds to gaps within scaffolds whose approximate sizes are known (Table S5 in Additional file 1). If we exclude scaffolds shorter than 1 kbp, the respective totals are 6.763 Gbp in contigs in 7.985 Gbp of scaffolds. Half of the assembly is represented in 304,023 contigs longer than 6.7 kbp and in 120,236 scaffolds longer than 21.2 kbp. (For scaffolds longer than 1 kbp the contig and scaffold N50 lengths are 8.3 kbp and 24.8 kbp, respectively.)

**Figure 2:**
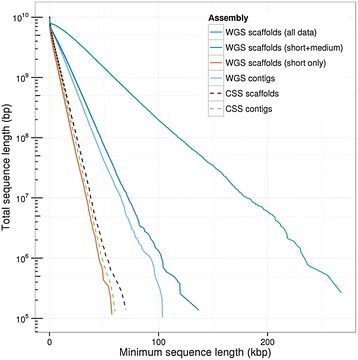
**Cumulative distributions of assembled sequence as a function of scaffold and contig length.** The total amount of assembled sequence in scaffolds or contigs longer than a minimum length is shown. As the available paired-end insert size is increased, the W7984 WGS assembly becomes progressively longer, with the inclusion of short-inserts (<500 bp) only (red); the addition of medium-inserts (700 bp to 1 kbp; dark blue); and finally the inclusion of approximately 4 kbp insert mate pairs (green). For comparison, the International Wheat Genome Sequencing Consortium chromosome-sorted assembly of ‘Chinese Spring’ (CSS) is also shown (black dashed line). Cumulative contig distributions for W7984 (light blue) and CSS (gray dashed line) are also depicted. As predicted by assembly theory, these quantities are exponentially distributed with decay lengths proportional to the N50 length scale of the assembly. This demonstrates that the excess length of the CSS assembly is restricted to an abundance of very short sequences (less than 1 kbp in length) that are outside of the body of the main exponential decay curves.

In comparison the chromosome-arm assemblies of ‘Chinese Spring’ [5] total 10.1 Gbp with a scaffold N50 length of 2.3 kbp excluding scaffolds shorter than 1 kbp; however, the total ‘Chinese Spring’ scaffold length drops to 7.0 Gbp with an N50 length of 4.2 kbp, so a full 3.1 Gb of this assembly is in very short scaffolds less than 1 kbp. Thus, our whole genome assembly using only short-insert data is comparable in quality to the chromosome-arm assemblies (also performed with only short-insert data, but typically with 30 to 200× shotgun depth compared with our uniform 28× short-insert coverage). When longer-range paired ends from a whole genome library are included, our WGS assemblies produce a substantially longer assembly, more than doubling the typical contig size and extending the scaffolding by a factor of 5 to 6 (Figure 2). As shown below, these extended sequences allow more complete genes to be captured, and enhances our ability to attach assembled scaffolds to the genetic map, and therefore to be positioned at a specific chromosomal location.

The high nucleotide-level accuracy of the WGS assembly is confirmed by comparison with six known genic sequences (exons plus introns) from the W7984 genotype: the three homeologs of the DELLA protein gene *Reduced height 1* (*Rht-1*) [32] and of the gibberellin biosynthesis enzyme *ent-kaurenoic oxidase* (*KAO*) [33]. These six genes provide 15,453 bp of known W7984 sequence; all are found to be contained within six scaffolds of at least 20 kbp in length with 11,043 bp (71.5%) covered by contig sequence from these scaffolds (an additional non-redundant 1,027 bp (6.6%) is found in five small scaffolds each less than 200 bp). While four discrepancies (Figure S2 in Additional file 1) were found between the assembly and the W7984 GenBank sequences, all four appear to be errors in the GenBank entries based on comparison with our shotgun sequence in accordance with prior assessment of the high base-level accuracy of meraculous [29,34]. When these same W7984 gene sequences are compared with the chromosome-sorted ‘Chinese Spring’ assemblies, one gene is not found, and the other five are captured across two or three scaffolds each, covering a comparable fraction of the genes (76.2% for Chinese Spring scaffolds of all sizes, versus 78.1% for our W7984 assembly).

**Figure 3:**
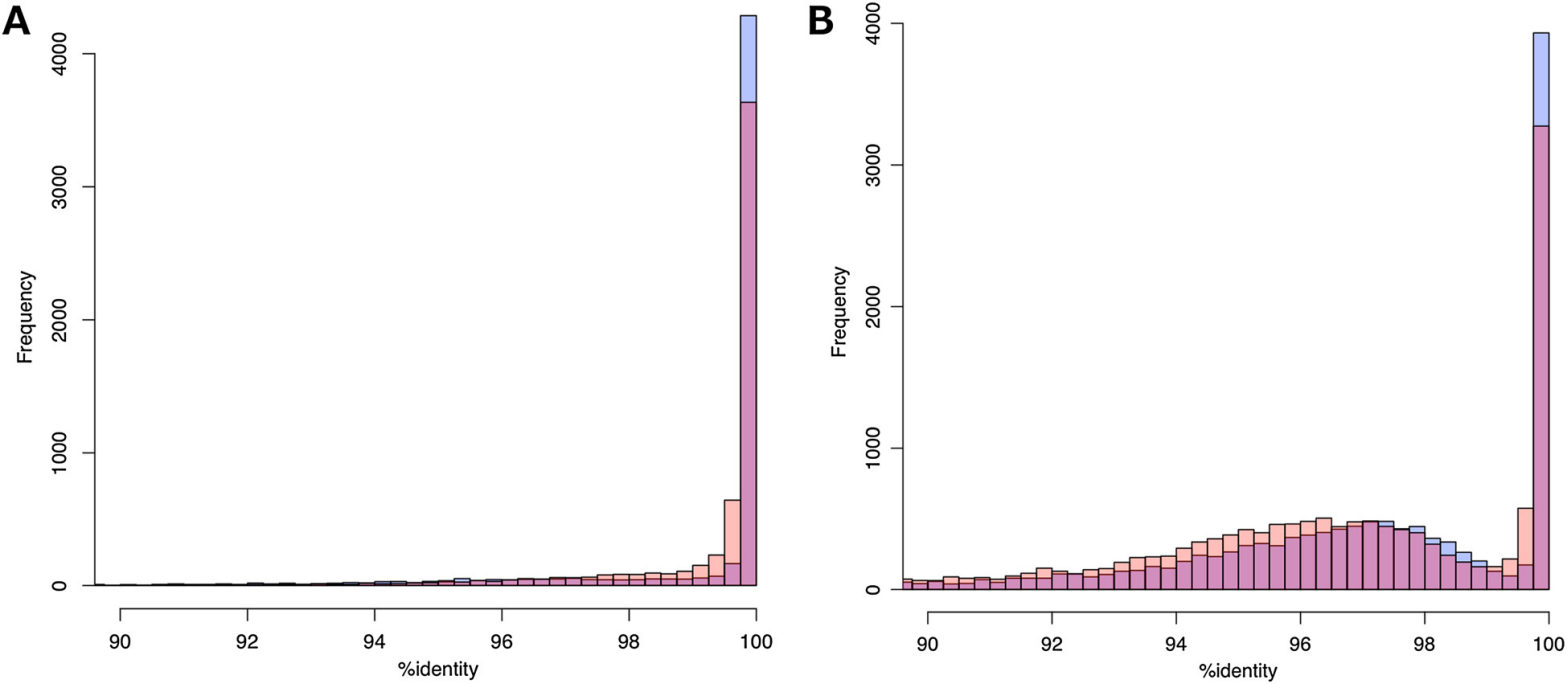
**Distribution of percent identities of alignments of ‘Chinese Spring’ full-length cDNAs versus genome assemblies. (A)** Frequency distribution of best percent identity of flcDNA alignments to IWGSC ‘Chinese Spring’ (blue bars) and W7984 WGS (red bars) assemblies. Results for both assemblies are superimposed; red and blue overlap is shown as purple. Included are all alignments longer than 50% of query flcDNA length. Note that while most ‘Chinese Spring’ cDNAs align at >99.75% identity to the IWGSC ‘Chinese Spring’ genome assembly, there is a long tail of lower identity best matches that could arise from errors in the genome assembly or in the flcDNA sequences. Matches to the W7984 assembly show most matches >99.50%, as expected given the intra-specific polymorphism between ‘Chinese Spring’ and W7984, but also show the long tail of lower identity. For W7984, these may arise from the absence in the genotype of the locus corresponding to the ‘Chinese Spring’ cDNA. **(B)** Frequency distribution of percent identity of flcDNA alignments longer than 50% of query flcDNA length, showing only those cDNAs with five or fewer such alignments. The secondary peak centered at approximately 97 to 97.5% corresponds to homeologous matches. As expected given the polymorphism between the two hexaploid wheat lines, the ‘Chinese Spring’ cDNAs align at slightly higher identity to their own genotype than to W7984.

To assess the global gene-space completeness of our whole genome assembly and the chromosome-sorted shotgun assemblies of the International Wheat Genome Sequencing Consortium (IWGSC), we compared them to a set of 6,000 (non-repetitive) full-length cDNA sequences from *T. aestivum* cv. ‘Chinese Spring’ [35] (Figure 3; Table S6 in Additional file 1). The majority of these cDNAs aligned over at least 50% of their length to single scaffolds in the two assemblies with the expected near-perfect identity (77.7% meraculous, 76.3% IWGSC; minimum 99% nucleotide identity). An additional approximately 20% are consistent with alignment to over 50% of the length of a homeologous locus with approximately 97% nucleotide identity (Figure 3B).

Our use of a 99% identity cutoff allows for intraspecific variation between W7984 and ‘Chinese Spring,’ and likely favors the chromosome arm shotgun assemblies, since they will match the ‘Chinese Spring’ cDNAs up to rare sequencing and assembly errors. While the SNP rate between these two lines is 0.38%, some loci will vary by more than 1% in W7984 and miss the cutoff, underestimating the completeness of our assembly. Conversely, loci that are missed by the ‘Chinese Spring’ chromosome shotgun assemblies may be credited with a ‘hit’ if a near-identical (99% identical) homeologous locus is aligned. We also note that it is likely, given the substantial presence/absence polymorphism observed in wheat and its close relative barley [36,37], that some of the Chinese Spring cDNAs represent loci that are absent in the divergent synthetic line W7984. The completeness of our assembly may, therefore, be underestimated by this approach. Interestingly, while 3,662 of 6,000 (61.0%) full length cDNAs are found at minimum 99% identity and 50% length in both assemblies, some cDNAs are found in one assembly but not the other, with a slight edge (1,001 versus 918) in our whole genome assembly (Figure S3 in Additional file 1).

These results demonstrate that the whole-genome assembly approach for wheat presented here is comparable in completeness to shotgun assemblies from sorted chromosomes, each capturing approximately three-quarters of known genes in reasonably complete form (more than half the transcribed sequence represented in a single scaffold). The gene spaces captured by the two approaches do not completely overlap, and thus have some complementarity to each other. Together the two assemblies capture 93% of known genes at the specified criteria of a minimum of 50% length covered at 99% identity. The WGS approach achieves longer-range linkage, however, due to the wider complement of mate-pair libraries.

### Ultradense genetic linkage map

To produce an ultra-dense genetic linkage map of hexaploid wheat, we used the POPSEQ [24] approach, generating low-depth WGS sampling of 90 DHs from the SynOpDH population (approximately 1.4× per individual). We used two complementary methods to discover segregating genetic markers, taking advantage of the abundant sequence variation between the parental lines (0.32% SNP rate). First, we aligned all reads to the *de novo* W7984 draft assembly and identified 24.6 million putative single nucleotide variants using standard methods. Since we required segregating SNPs for mapping, we eliminated variants that were due to homologous/paralogous alignment and sequencing error by filtering the candidate variants based on expected allele frequency for a bi-parental DH population. Filtering reduced the putative variants to 19.0 million robustly segregating SNPs that were subsequently used for genetic mapping and anchoring.

In a second, assembly-independent approach, we identified 2.2 million pairs of 51-mers that (1) share a common 50-mer prefix, differing only in their final base (polymorphic condition); (2) are the only 51-mers with this 50-mer prefix (bi-allelic condition); (3) are found differentially in the parental data sets (polymorphic condition); (4) are each found in a narrow frequency range (40 to 50×) in the pooled SynOpDH data (approximately 90× homozygous 51-mer depth) (segregation condition). These pairs represent 50-mers that occur at single copy in both W7984 and Opata, but where the 51st nucleotide differs in the two parents due to allelic polymorphism (SNPs or other variants). After eliminating 51-mer pairs that occurred in both allelic states in any DH individual, we find 1.7 million remaining pairs that behave as segregating markers in the SynOpDH population. We observed a low level of sequencing error, residual polymorphism and/or cross-sample contamination. The number of segregating variants obtained by both of our approaches exceeds the number of markers used in recent sequence-based genetic mapping efforts [38-40] by three orders of magnitude.

**Figure 4:**
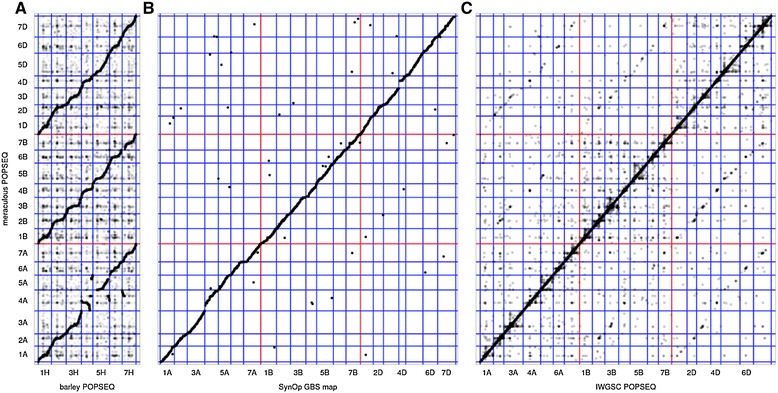
**Validation of the POPSEQ genetic map. (A)** POPSEQ positions [24] of barley high-confidence genes [45] were compared with the genetic positions of their putative orthologs in our wheat POPSEQ map. Assignment of orthologous groups agreed in 87% of the cases. Genetic positions within the orthologous group showed high collinearity (Spearman’s ρ = 0.936). Known translocation events relative to barley involving wheat chromosomes 4A, 5A and 7B [46] could be traced with high precision. **(B)** Collinearity with a previous genetic map of the Synthetic × Opata population constructed through genotyping by sequencing [28]. A total of 11,000 out of 20,000 genotyping-by-sequencing tags carrying SNPs could be uniquely mapped to our assembly. Chromosome assignments agreed for 99.5% of the genotyping-by-sequencing tags aligned to anchored sequence scaffolds. Genetic positions within linkage groups were highly correlated (Spearman’s ρ = 0.995). **(C)** Chromosome shotgun contigs were anchored to the same genetic framework as the meraculous scaffolds of W7984. Genetic positions of contigs and scaffolds matched by sequence alignment differed by less than 5 cM in 99.1% of the cases. Chromosomes are separated by blue lines, subgenomes by red lines.

The markers were clustered into linkage groups using log-odds (LOD) score thresholds by two methods, including a new, computationally efficient clustering algorithm that exploits the inherent linearity of genetic maps [41]. From the 21 resulting clusters, we subsampled robust markers with little or no missing data to build a framework genetic map using standard software [42]. Preliminary linkage maps identified 10 SynOpDH individuals with partial or complete loss of a chromosome arm, which were excluded from the final map construction. Scaffolds with co-segregating SNPs were then anchored to map locations based on a LOD score >8. Using a second iterative approach, a high confidence framework map with minimal missing data was produced using 112,687 markers and totaling 2,826 cM in 1,335 recombination bins (Table S7 in Additional file 1). As expected for a DH population, some regions of the genome showed segregation distortion [43,44] (Figure S4 in Additional file 1) with a bias for either Opata (on 6AS and 6DS) or for W7984 (4DL). Shotgun sequence-based maps made with the two independent approaches show near perfect agreement. For example, of scaffolds placed on the map by both methods, only 0.002% are discordant with respect to chromosome identity, and map coordinates between the two methods are correlated (with a Pearson r-value of 0.95) and with an independently generated genetic map [28] (Figure 4B).

### Integration and validation

Our final integrated sequence map assigns a large fraction of the assembly and the transcribed genes to chromosomal locations (Table 1). It incorporates 78.02% of the total assembled scaffold length (7.113 Gbp), and 94.89% of the assembled length in scaffolds at least 10 kbp long (235,647 out of 253,986 scaffolds). We compared the positions of barley gene models anchored to an ultra-dense map of the barley genome [24] to the positions of their wheat orthologs (Figure 4A). Orthologous group assignments were largely concordant (87%) and collinearity within groups was very strong (Spearman’s ρ = 0.936). Similarly, we found near-perfect collinearity between the genetic positions of meraculous scaffolds and IWGSC contigs that were anchored to the same genetic framework map (Figure 4C).

**Table 1:** **Summary of assembly and anchoring statistics**

**Assembly**	**W7984 (WGS, this report)**	**Chinese Spring (chromosome sorted shotgun, IWGSC 2014)**
Scaffolds ≥1kbp	645,811	2,272,234
	8.00 Gbp	7.05 Gbp
Map-anchored scaffolds ≥1 kbp (percentage of total assembled base pairs)	437,973	1,175,794
	7.13 Gbp (89.3%)	4.46 Gbp (63.2%)
Scaffolds ≥10 kbp	253,986	91,141
	6.55 Gbp	1.31 Gbp
Map-anchored scaffolds ≥10 kbp (percentage of total assembled base pairs)	235,647	74,520
	6.21 Gbp (94.9%)	1.08 Gbp (82.3%)
Full-length cDNAs captured on the assembly (at least 50% length; out of 6,000)	4,663 (77.7%)	4,580 (76.3%)
(minimum length 25%)	5,288 (88.1%)	5,428 (90.5%)
Full-length cDNAs placed on map-anchored scaffolds (at least 50% length)	4,353 (72.6%)	3,404 (56.7%)
Full-length cDNAs placed on map-anchored scaffolds (at least 25% length)	4,863 (81.1%)	3,909 (65.2%)
Concordance of POPSEQ positions	99.4%	

This table provides a comparison between the POPSEQ anchored assemblies of W7984 and ‘Chinese Spring’ using a chromosome sorting and WGS approach, respectively. Shown are total scaffolds (minimum length 1 kbp), total map-anchored scaffolds, and capture of known full-length wheat cDNAs on the assemblies both before and after chromosome anchoring. The final row shows concordance as measured by the percentage of pairs of anchored chromosome shotgun contigs and WGS scaffolds that were matched by sequence alignment and were genetically positioned within 5 cM of each other. ‘Chinese Spring’ and WGS scaffolds are paired if there is megablast hit with ≥99% identity and ≥2,000 bp alignment length between them. Only the best hit of each ‘Chinese Spring’ scaffold was considered.

Of the 6,000 non-transposon-related full-length cDNAs from ‘Chinese Spring’, 72.6% could be aligned to the integrated W7984 sequence map over 50% of their length. This is substantially more than the 56.7% of full-length cDNAs that can be assigned to contigs of the chromosome-arm shotgun assemblies [5] anchored to the genetic framework map using the same criteria. With a weaker restriction of 25% length alignment, our map-anchored assembly captures 81.1% of known genes, while the map anchored chromosome-arm shotgun assemblies capture only 65.2%. This is consistent with the high degree of fragmentation of the chromosome-arm assemblies based on only a single insert library, which limits both their ability to capture entire genes as well as their ability to be placed on the genetic map. Note that by using independently known full-length cDNAs, our comparative analysis of the assemblies is independent of the completeness or quality of the predicted IWGSC gene set. Lists of cDNAs that can be found with ≥99% in only one of the assemblies are given in Additional files 2 and 3.

The ultra-dense genetic map also allowed us to validate the local accuracy of our WGS assembly, since SNP markers at the ends of an assembled scaffold should show identical (or occasionally almost identical) segregation patterns and therefore lie at the same map position. Discrepant segregation of markers at the ends of a scaffold therefore suggests an assembly error internal to the scaffold. By this approach, we estimated that the mis-join rate of the WGS assembly is approximately one per 1,000 scaffolds (or less than one mis-join per 3.2 Mbp of scaffold sequence). IWGSC contigs assigned by sequence alignment to the same meraculous scaffold had concordant chromosome assignments in 99.6% of the cases, further supporting the high accuracy of our scaffolding algorithm. The limited discrepancies can arise from mis-assembly in our whole genome approach, mis-sorting in the chromosome-based strategy, or mis-identification of homologous scaffolds between the two wheat genomes (based on 99% identity, 2 kbp length).

### Diversity between wheat accessions and subgenomes

**Figure 5:**
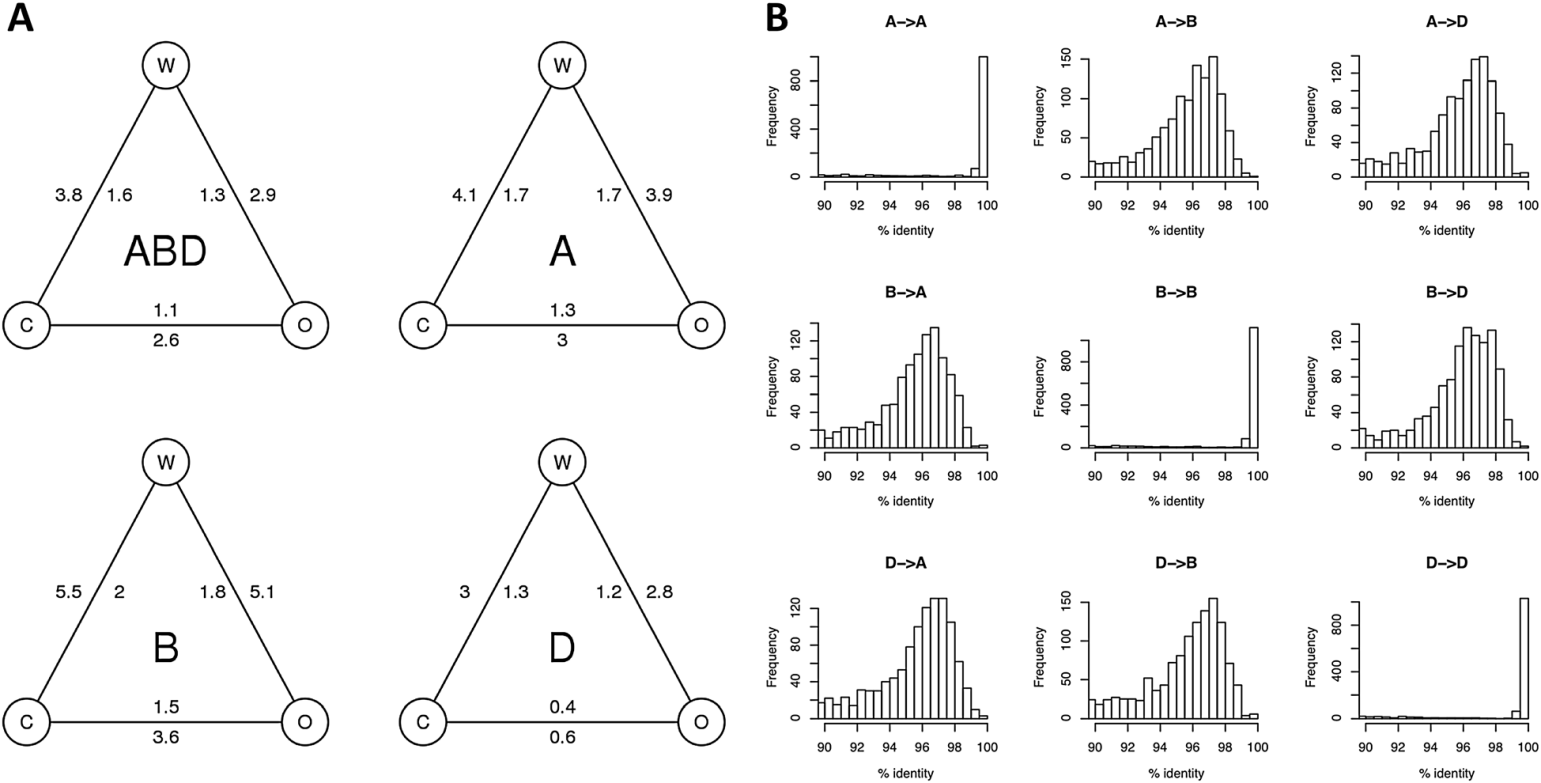
**Nucleotide diversity in the wheat genome. (A)** The average number of SNPs per kilobase between the three wheat types Chinese Spring (C), Opata (O) and W7984 (W) is shown across all three subgenomes (ABD) or in the individual subgenomes (A, B and D). The numbers on the outside of the triangles gives the diversity across all sequences in the respective subgenomes, those on the inside give the diversity in coding sequences only. **(B)** Diversity between homeologous genes. Full-length cDNAs [35] were aligned to our assembly of W7984 and assigned to one of the subgenomes using the genetic anchoring of the assembly. This plot shows the distribution of nucleotide identity between cDNAs assigned to the A, B and D subgenomes and their best BLAST hit in the other two subgenomes (that is, to their putative homeologous loci).

We used our alignments of short reads of Opata and W7984 against the assembled sequence of Chinese Spring [5] to estimate the nucleotide diversity between these three genotypes (Figure 5A). The diversity in coding sequences was slightly less than half that of the entire genome. There were fewer differences between the two wheat cultivars Chinese Spring and Opata M85 than between either of these and the recently synthesized W7984. This trend is most pronounced in the D genome, which has lost a large fraction of the diversity found in the progenitor genome of *Aegilops tauschii* [47]. The reduced diversity in the D genome of *T. aestivum* cultivars has been an obstacle to genetic map construction in mapping populations derived from elite breeding material [48], but can be overcome by using synthetic wheats such as W7984. We note that SNP rates based on short read alignment may be underestimates because short reads originating from regions of high diversity are more difficult to align to a diverged reference. For instance, the SNP rate between W7984 and Opata M85 based on alignment to the assembly of W7984 (0.32%) is higher than the rate calculated from alignments against Chinese Spring (0.29%).

In addition to SNPs, we also searched for larger deletions in W7984 relative to Chinese Spring and Opata M85. We found 1,501,127 intervals ≥50 bp (cumulative length: 343.0 Mb) that were present in the assembly of Chinese Spring and were covered by Opata M85 reads, but had no read coverage in W7984 (Dataset S1 in Additional file 4). Relating the cumulative length of all deletions in a subgenome to the length of all genetically anchored contigs, we found that 1.17%, 1.19%, and 1.07% of the anchored sequence of the A, B and D subgenomes, respectively, exhibited presence-absence variation between W7984 and Chinese Spring. However, only 15.9% of deleted intervals (54.7 Mb) were located on genetically anchored (that is, mostly low-copy) regions. This finding supports the notion that presence-absence variation is common in the highly repetitive genome of polyploid wheat.

Lastly, we used our alignments of cDNA sequences against the assemblies of W7984 and Chinese Spring to estimate the diversity between the three subgenomes of hexaploid wheat. The three subgenomes were clearly differentiated (Figure 5B). The identity of full-length cDNAs to their best BLAST hits, that is, their true positions in one of the subgenomes, was >99% in the majority of cases, whereas the identity to their second best hit, that is, a homeologous locus in one of the other subgenomes, was only approximately 97%.

## Discussion

We have produced a genetically anchored WGS assembly of the hexaploid wheat genome. This shotgun assembly captures more than three-quarters of known wheat genes, and the ultradense genetic map anchors over 81.1% of the transcribed genes to a chromosomal position. Remarkably, the hexaploid structure of the bread wheat genome was not an insurmountable obstacle for a WGS approach, since we could exploit the sequence divergence between sub-genomes and disomic inheritance in bread wheat.

Recently, the IWGSC has published shotgun assemblies of 40 chromosome arms and the complete chromosome 3B of bread wheat that were constructed only from a single type of short-insert paired-end library, since it is generally not possible to construct useful long-insert mate-pair libraries from DNA of flow-sorted chromosomes that have been subjected to multiple displacement amplification [49]. Compared with a chromosome-by-chromosome shotgun approach, our WGS approach has the apparent disadvantage of having to disentangle homeologous regions from the three subgenomes. However, this drawback is more than offset by the ability to use long-range connectivity information afforded by easily constructed mate-paired libraries.

An intuitive explanation for this result is that chromosome sorting only simplifies the separation of homeologous sequences in genic or low-copy regions. By contrast, the most common transposable elements occur so abundantly even in only a single chromosome arm that they thwart attempts to assemble them correctly with short reads only. In light of this limited utility of a chromosome-by-chromosome shotgun approach, ongoing and future genome sequencing projects in other highly repetitive and/or polyploid cereal crops, such as rye and oats, may adopt a simpler, straight-forward whole-genome strategy to construct a draft sequence assembly instead of establishing elaborate protocols for efficient flow-sorting and subsequent chromosome-wise shotgun assembly. Likewise, it may be feasible to construct a genome-wide physical map of the wheat genome using sequence-based fingerprinting methods [50] that can distinguish between fragments from homeologous loci. These considerations do not diminish the importance of clone-based approaches to achieving the ultimate goal of a finished sequence for hexaploid wheat, the long-term aim of the IWGSC [51].

At first glance, the summary statistics of our assembly might look unimpressive. After all, we were able to assemble only 9.1 Gbp of a total estimated genome size of 16 Gbp. However, the fraction of the genome in assembled contigs is in the same range as the chromosome-by-chromosome shotgun assembly of IWGSC [5] (9.1 Gbp versus 10.1 Gbp), suggesting that the problems are intrinsic to the wheat genome and short read datasets. Importantly, the better contiguity of our assembly made it possible to anchor a much larger fraction of the genome (7.1 Gbp versus 4.4 Gbp) to chromosomal locations using the same genetic information that was used to anchor the IWGSC assembly, but taking advantage of longer scaffolds that have a higher probability to carry at least one segregating polymorphism. Moreover, our assembly is substantially better than a first WGS assembly of hexaploid wheat from 5× coverage of 454 reads [4]. The N50 of this assembly was far below 1 kb and it was only with the help of an additional transcriptome assembly that complete gene sequences could be constructed and at least partially assigned to one of the subgenomes. If we seek comparison outside the Triticeae, the contiguity and genome representation of our assembly are worse than those of a WGS assembly of white spruce, which achieved an N50 of approximately 20 kb and near-complete genome coverage [14]. However, the repeat structure of conifer genomes may be less adverse to WGS assembly than that of cereal grasses, since the genome of loblolly pine was found to contain fewer nearly identical repetitive elements than the genome of maize or sorghum [52].

Despite its obvious shortcomings, our assembly will serve as a useful resource for the wheat community, very much like the incomplete and highly fragmented assembly of barley, which nevertheless has enabled the development of cost-efficient resequencing strategies [53], reference-based genetic mapping [54] and fast gene isolation [55]. Integrating the WGS assembly of barley with a genome-wide physical map, clone sequence information and gene models predicted from RNA sequencing resulted in a highly useful genomic framework of the barley genome [45], mapping 1.2 Gb of largely genic sequences. The sequence resources and genetic marker information provided by the present wheat assembly will assist the ongoing efforts of producing at first physical maps and then map-based sequences of all chromosome arms of wheat. So far, these efforts had to rely on the barley POPSEQ map as a proxy [56] or low-density conventional maps that are difficult to integrate with scarce sequence data [57].

Even in the context of WGS methods, our assembly can still be improved. The addition of more shotgun sequence depth would allow longer k-mers to be used, resulting in the incorporation of more repetitive sequences. It is worth emphasizing that while the wheat genome is commonly described as being 80% repetitive [58], this is a biological criterion based on transposable element detection and classification. Depending on the choice of k, far more than 20% of the genome is accessible to shotgun assembly, since diverged ‘repetitive’ sequences can still be distinguished at the nucleotide level. Even with our modest choice of k = 51, more than 40% of the hexaploid wheat genome can be assembled and mapped. We also note that the shotgun coverage of the recombinant progeny accounts for a substantial amount of sequence that could, in principle, be incorporated into the assembly with further algorithm development. Inclusion of longer-insert mate pair sequences (for example, fosmids and bacterial artificial chromosomes (BACs) [59]), and integration with long reads and optical maps can further improve scaffolding and better organize sequence within genetic bins, which can themselves be partitioned simply through the addition of more recombinant progeny sequenced at low coverage.

## Conclusions

Our method provides a straightforward approach to tackling large and complex (as well as simple) genomes using straightforward WGS methods.

## Materials and methods

### Biological material

Hexaploid wheat (for example, ‘bread’ or ‘common’ wheat) formed around 8,000 years ago through a natural hybridization between cultivated tetraploid wheat (AABB genome) and a wild wheat relative, *Ae. tauschii* (DD genome) [60]. Commonly known as bread wheat, the hexaploid species is widely cultivated throughout the world. The tetraploid wheat species (also referred to as ‘Durum’ or ‘pasta’ wheat) represents an older group of wild and cultivated material. Durum wheat is the modern form of a 10-millenia aged crop complex represented by various taxa of the same *Triticum turgidum* spp. Durum wheat (*Triticum turgidum* ssp. *turgidum* var. *durum* (Desf) Husn.) is represented by landraces and elite inbred lines. *T. turgidum* is domesticated from wild emmer (*Triticum turgidum* ssp. *dicoccoides*) and is allotetraploid (2n = 4x = 28, genomes AABB). Durum wheat is a selfing species and commercial varieties are mostly pure lines. The diploid D genome species, *Ae. tauschii*, is a wild annual grass native throughout central Asia.

‘Synthetic W7984’ is a contemporary reconstitution of hexaploid wheat formed by hybridizing a tetraploid wheat *Triticum turgidum* L. subsp. *durum* var ‘Altar 84’ (AABB genotype) with the diploid goat grass *Ae. tauschii* (219; CIGM86.940) (DD genotype). Following chromosome doubling, this synthetic hexaploid is interfertile with bread wheat and is typically regarded as a variety of *T. aestivum*.

*T. aestivum* var ‘Opata M85’ is a hexaploid bread wheat cultivar developed in the wheat breeding program at the International Wheat and Maize Research Center (CIMMYT). It is a medium quality, medium maturity hard white spring wheat.

Synthetic W7984 and Opata M85 are parents of the widely used DH genetic reference population ‘SynOpDH’ [27]. For this population a total of 215 DH lines were produced from two F1 plants. The F1s were made from a cross between two single plants using W7984 as female and Opata as male. From the parental cross, two F_1_ plants were used to form the DH lines using the maize pollinator method [27].

Seeds for the Synthetic W7984, Opata M85 accessions and SynOpDH lines used in this study can be obtained upon request from the Wheat Genetics Resource Center at Kansas State University.

### Shotgun sequencing of the synthetic wheat W7984

WGS Illumina libraries were prepared using DNA isolated from etiolated seedlings. For each of the parental lines, tissue from a minimum of 20 plants was sampled and pooled together for DNA extraction. A standard CTAB (cetyltrimethyl ammonium bromide) extraction was used with RNase treatment. For DH lines, six seedlings were sampled and DNA was extracted using the Qiagen BioSprint 96 Plant DNA extraction kits and robot. TruSeq Illumina fragment libraries of size approximately 250 bp and approximately 500 bp were sequenced using 2×150 chemistry on a HiSeq 2000 instruments. A summary of the dataset can be found in Table S1 in Additional file 1. Three ‘800 bp’ fragment libraries were prepared and sequenced using long run chemistry on the Illumina HiSeq 2500, producing nominal paired 250 bp reads. Two of the three attempted ‘800 bp’ libraries showed substantial bimodality when aligned to preliminary assemblies, including not only the desired peak insert size at approximately 800 bp but also a large collection of pairs with short inserts (<400 bp). All sequences were used for contig building, but only unimodal libraries were used for scaffolding. Two LFPE (ligation-free paired end) mate pair libraries were generated as follows. DNA fragments were generated using the 5500 SOLiD Mate-Paired Library Construction Kit (SOLiD®). Genomic DNA (5 μg) was sheared using the Covaris E210 (Covaris (Woburn, MA, USA)) and gel size selected to target an insert size of 1.5 kbp (library OAGT) and 4 kbp (PSWH). The sheared DNA was end repaired, and ligated with biotinylated internal linkers. The DNA was then circularized using intra-molecular hybridization of the internal linkers. The circularized DNA was treated with plasmid safe to remove non-circularized products. The circularized DNA was nick translated and treated with T7 exonuclease and S1 nuclease to generate fragments containing internal linkers with genomic tags on each end. The mate pair fragments were A-tailed and purified using Streptavidin bead selection (Invitrogen). The purified fragments were ligated with Illumina adaptors and amplified using 10 cycles of PCR with Illumina primers to generate the final library. Quantitative PCR was used to determine the concentration of the libraries and were sequenced on the Illumina Hiseq. The distribution of insert sizes is measured to be approximately 1.0 kbp for OAGT and approximately 4.2 kbp for PSWH (Figure S5 in Additional file 1).

### Sequencing of *T. aestivum* ‘Opata M85’ and the SynOpDH population

To identify variants that differentiate Synthetic W7984 from Opata M85, we produced approximately 19× shotgun coverage for Opata M85 (Table S2 in Additional file 1). Since *de novo* assembly was not our aim, no mate pairs were generated. 51-mer depth is shown in Figure 1. Note that each read of length R is tiled by R - k + 1 k-mers, and each sequencing error affects k k-mers and therefore the k-mer depth is reduced by approximately ke/2, where e is the per base error rate and the factor of ½ roughly accounts for the fact that most errors occur near the end of a read. Thus, although the raw shotgun coverage is approximately 19×, the peak 51-mer frequency is approximately 11 × .

### *De novo* whole genome assembly

Assembly was performed using meraculous [29] and is available for download [61]. The Perl code used to perform the assembly is available online [62] (with exceptions noted below). Several modifications to the core meraculous code-base were made to improve the performance of the assembler for this data set. These modifications are available for download [63].

The primary purpose of these code variants is to more fully take advantage of long (251 bp) reads in the assembly when the initial ‘UU’ contig generation procedure yields a highly fragmented preliminary result. In addition, a high-performance parallel version of the contig-generating k-mer-graph traversal phase of the assembly was developed with Unified Parallel C (UPC) and run on the NERSC Edison supercomputer (a Cray XC30) saving several days of compute time over the standard Perl implementation [64]. This high-performance implementation is based on a distributed hash table employing communication optimizations. We also leverage a lightweight synchronization scheme that relies on a state machine. De Bruijn graph traversal along uncontested ‘UU’ paths [29] took approximately 110 seconds on 3,072 cores or approximately 67 seconds on 6,144 cores. This code is available upon request.

The assembly was performed using an initial k-mer length of 51 (parameter -m = 51) and minimum k-mer frequency of three (parameter -D = 3). Contigs were generated using all short fragment libraries (but excluding mate-pair libraries). An initial round of scaffolding was performed using reads from all fragment libraries that were found to ‘splint’ pairs of contigs by 51-mer alignment. This splint-only-scaffolding protocol has not been used in previous meraculous assemblies, but was developed specifically to cope with the unique combination of insert sizes, depths of coverage, and genome complexity presented by this project. A minimum of three splinting alignments was required to accept a scaffolding link at this stage (parameter -p = 3).

Three additional rounds of scaffolding were performed following standard meraculous protocol for short (200 to 500 bp), medium (700 to 1000 bp), and long (4 kbp) libraries, each using a minimum of two spanning-pair alignments to accept a scaffolding link (parameter -p = 2). For the mate-pair libraries (OAGT, PSWH) reverse complementation and 3′ truncation (parameters -R, −U 3, respectively) were used to accommodate these library types. Additionally, short-pair elimination (parameter -D 600) was used for the UAXO library to deal with its moderate bi-modality, and the library H0036 was entirely excluded from this form of scaffolding due to extreme bimodality (Figure S5 in Additional file 1). Finally, gap-closing was performed using optional parameters -A, −D = 3, −R = 1.75.

With the exception of the contig-generation phase noted above, computations were performed on the JGI Genepool system (a 450-node sub-cluster with eight 48Gb, Intel Xeon L5520 2.27 Ghz cores per node and a dedicated 32-core 500 Gb SMP (Symmetric MultiProcessing) node were used). The k-mer counting and graph-generation steps required 5.6 k core-hours across 288 jobs. The read-alignment phase required a total of 30.8 k core-hours across 8.4 k jobs. The gap-closure phase required 3.5 k core-hours across 2.8 k jobs. These phases represent the vast majority of the computational resources required.

### Contaminant screening of the assembly

Chloroplast, mitochondrial, prokaryotic, and fungal contaminants were sought by aligning the wheat scaffolds using blastx (parameters: −p blastx -a 7 -Q 11 -f 12 -W 3 -F ‘m S’ -U -e 1 -m 8 -b 10000 -v 10000) against the NCBI non-redundant proteins [65] for each category as the database. Ribosomal DNA was identified using megablast (parameters: −a 7 -b 0 -f T -D 3) against the NCBI non-redundant rDNA set. All alignments were initially filtered for a bit score ≥300, and scaffolds indicating a significant alignment were classified into bins. A total of 17,054 scaffolds (26.8 Mbp) were identified as likely contaminants, with 5,766 mitochondrion (5.6 Mbp), 451 chloroplast (338 kbp), and 10,837 prokaryote (21 Mbp). Contaminants included known sequencing-related microbial contamination, including *Delftia* spp. and *Stenotrophomonas* spp., but not obvious microbial or fungal commensals or pathogens associated with wheat. All subsequent analyses of the assembly excluded these contaminant scaffolds, unless otherwise noted.

### Validation of assembly versus known transcripts and completeness relative to known transcribed genes

To assess the completeness of the genome assembly with respect to known transcribed sequence, we used a collection of 6,137 flcDNAs in the ‘Triticeae full length cDNA database’ [66] from *T. aestivum* var ‘Chinese Spring’ generated by Mochida *et al*. [35]. These flcDNAs are from hexaploid bread wheat and are expected to match our W7984 assembly with the exception of intra-specific polymorphisms and presence/absence or copy number variation. In contrast, they are expected to match the IWGSC ‘Chinese Spring’ assemblies identically. We used flcDNA rather than short-read RNAseq because the cDNA data are longer, of higher quality, and as clones are not subject to confounding effects arising from attempting to assemble homeologs in distinct scaffolds. We cleaned the flcDNAs by (1) trimming polyA tails with BioPerl ‘TrimEST’; (2) identifying non-wheat contaminations, using BLAST [67]; and (3) identifying putative transposable elements by comparison with RepBase [68].

#### Contamination

We identified three *T. aestivum* flcDNAs in GenBank as being in fact human sequences (RFL_Contig2039, 3209, and 5006) showing near 100% identity to human genes. These are presumably low-level contaminants of the wheat cDNA libraries. These sequences were excluded from further consideration.

#### Transposable elements

We found 99 *T. aestivum* flcDNAs from the Mochida *et al*. set (99/6,137 = 1.6%) with substantial BLAST alignments (BLASTN default word size, e-10, no DUST filter; >90% identity over >50% of their length) to RepBase entries. These were considered to be transposable elements and not considered in subsequent analyses.

#### Putative non-wheat sequences

To identify other likely non-wheat contaminations in Mochida *et al*. [35], we used BLASTN (e-10, no DUST filter; >90%) versus the GenBank non-redundant nucleotide database, and excluded from further consideration flcDNA sequences that (a) had no alignment to both our W7984 assembly and the ‘Chinese Spring’ assembly (>80% length, 1e-10) and (b) did not hit grass sequences in GenBank (>90% identity, >10% length). We found 52 flcDNA sequences that did not align to either assembly. Of these, 17 had alignments to grasses and were kept in further analyses; 32 had no GenBank hits to plants; 3 had only weak hits to non-grasses. These last two categories were not considered further.

Thus, after filtering for contaminants and transposons we consider 6,000 known, non-transposon *T. aestivum* flcDNAs = (6,137 initial flcDNA from Mochida *et al*.) - (99 RepBase transposon-related) - (3 human contamination) - (35 likely non-grass contamination not found in either assembly).

We also identified flcDNAs that have 10 or more alignments (>80% identity, >50% length) to one or both of the hexaploid wheat assemblies (126 to W7984, 198 to ‘Chinese Spring’). These are also likely to be repetitive elements, but may include recently diverged large gene families. These are included in all analyses.

#### Alignment to W7984 and ‘Chinese Spring’ assemblies

Non-transposon, non-contaminant cDNA sequences were aligned to both the meraculous W7984 WGS assembly database and to the IWGSC chromosome sorted ‘Chinese Spring’ assembly database with BLAST (BLASTN default word size, e-10, no DUST filter), initially requiring >80% identity over >50% of the cDNA or mRNA length. The high-scoring pairs (HSPs) of cDNAs aligned to genomic sequence correspond to exons, and minimally overlapping HSPs to a given scaffold were combined to produce a single percentage coverage (Total bases aligned/Total bases in cDNA) and percentage identity (Total positions matched/Total aligned positions excluding gaps).

### Shotgun sequencing-based genotyping of the SynOpDH population

To genotype the SynOpDH mapping population we lightly shotgun sequenced 90 individuals. All sequencing was from unamplified fragment libraries nominally with 500 bp inserts, with 2×150 paired-end Illumina reads run on the HiSeq2000. Of these, three samples had less than 1× coverage, with the remaining samples having 1 to 2× read coverage (median: 1.38×, mean 1.37×, standard deviation 0.20×). (The estimated coverage was computed by dividing the total number of base pairs by 17 Gbp, without any attempted correction for contamination, adapters, and so on.)

A data summary is provided in Table S3 in Additional file 1. Briefly, sequences were indexed and pooled using Illumina TruSeq with indices as specified in Table S3 in Additional file 1. Estimated read depth is based on total sequence (Number of raw reads × Read length) divided by an estimated genome size of 17 Gbp. It does not include any correction for organellar contamination or artifacts. The ‘% artifact’ was estimated from 1% of reads; it was based on k-mer matches to a database of known sequencing artifacts at JGI. The ‘% organelle’ is estimated by comparing reads to the mtDNA and cpDNA of wheat.

The k-mer frequency distribution for the pooled reads of the mapping population is shown in Figure S7 in Additional file 1.

Note: SynOpDH IDs 0010, 0019, 0026, 0028, 0033, 0034, and 0117 were found to have deletions in chromosome 2D, 0031 in chromosome 3B, and 0083 in chromosome 7D. IDs 0030 and 0118 were found to have high rates of heterozygous markers, which is attributed to contamination. Data for these IDs were excluded from consideration in building the framework map.

### Genetic map construction with POPSEQ

#### Read mapping and SNP calling

Shotgun sequence reads were mapped against all contigs ≥1 kbp of the meraculous W7984 WGS assembly using BWA-MEM version 0.7.7 [69]. Sorting of BAM files and duplicate removal were performed with PicardTools 1.100 [70]. SNPs and genotypes were called with the samtools mpileup/bcftools pipeline (version 0.1.19) [71]. The parameters ‘-B’ and ‘-D’ were supplied to samtools mpileup to disable BAQ calculation and record per-sample read depth. Genotype calls were filtered and converted into genotype matrix with an AWK script (available as Text S3 of Mascher *et al*. [54]). SNP calls with quality scores below 40, more than 90% missing data, or a minor allele frequency below 5% were discarded. The full genotype matrix is available as Dataset S2 in Additional file 4. The same procedures were also performed to produce a genotype matrix from the results of read mapping and SNP calling against the IWGSC assembly of cv. Chinese Spring [5].

#### Framework map construction

High-quality consensus genotypes were constructed for the meraculous scaffolds similar to the method described by Mascher *et al*. [24]. Only SNP positions at which both parents had successful genotype calls and were homozygous for opposite alleles were considered. Heterozygous calls in the DH progeny were set to missing. At least three successful genotype calls per individual and 95% concordance across all SNP positions on a scaffold were required to assign a scaffold genotype to an individual. Scaffold consensus genotypes with at least 10 genotype calls for each of the two parental alleles and less than four missing calls in the progeny were used as potential framework markers. The Hamming distance between all pairs of framework markers was calculated with a C program [24]. Groups of markers with pairwise Hamming distance 0 were put into the same bin of markers and the only the marker with the fewest number of missing genotype calls was selected as the representative of the bin. A total of 1,335 bin representatives were used as input for genetic map construction with MSTMap [42]. MSTMap was called with the following parameters: population_type DH, distance_function kosambi, cutoff_p_value 0.0000005, objective_function ML. All input bins were clustered in one of 21 linkage groups corresponding to the 21 chromosomes of wheat and positioned at distinct genetic positions in the output of MSTMap. The final map length was 2,826 cM. The genetic positions of framework markers are available as Dataset S3 in Additional file 4. Preliminary maps indicated the presence of large-scale deletions encompassing entire chromosome arms in 10 of the 90 DH lines. Additionally, two individuals showed an excess of heterozygous calls. These individuals were not used for map construction. Thus, the final framework map was made with genotypic data from 78 DH lines.

#### Anchoring scaffolds onto the framework map

Scaffolds of the meraculous assembly were placed into the framework map by finding the nearest neighboring genotype vectors in the set of framework markers as described by Mascher *et al*. [24]. Scaffold consensus genotypes were constructed as described above, but only a single successful genotype call per scaffold was required. Consensus genotypes with more than 70% missing calls were discarded. Nearest neighbor search was done with a C program [24]. Scaffold consensus genotypes having a Hamming distance >3 to their nearest neighbor(s) were discarded. If a scaffold had more than one nearest neighbor, we required ≥90% of the markers to come from the same chromosome and the median absolute deviation of genetic positions to be ≤5 cM. The genetic positions of scaffolds are available as Dataset S4 in Additional file 4. The same procedures were used to place IWGSC contigs onto our framework map. The genetic positions of contigs of the Chinsese Spring are available as Dataset S5 in Additional file 4.

#### Comparison to other datasets

All contigs ≥1 kbp of the IWGSC assembly of cv. Chinese Spring were aligned against all meraculous scaffolds of W7984 with megablast [72]. Only HSPs longer than 500 bp and sequence identity ≥98.5% were considered. The longest HSP of each IWGSC contig was used to assign it to a meraculous scaffold. Sequences of 64 bp genotyping-by-sequencing tags mapped previously in the Synthetic W7984 x Opata M85 DH population [28] were aligned to the meraculous assembly of W7984 with BWA-MEM (version 0.7.7) [69]. Only tags with the best possible mapping score (uniqueness) of 60 were retained. Coding sequences of barley high-confidence genes [45] were aligned to meraculous scaffolds using BLASTN [73] considering only hits with identity ≥90% and alignment length ≥200. Genetic positions of barley genes were taken from Mascher *et al*. [24]. Genetic positions of different maps were compared against each other and plotted with standard functions of the R statistical environment [74].

### K-mer based genetic map

#### Defining 50 + 1-mer markers

A high-performance k-mer counting algorithm [64] was developed and used to count 51-mer frequencies in each of the two parental fragment data sets as well as the pooled SynOpDH population data. Using 9,600 cores of the NERSC Edison system, this counting was performed in less than 30 minutes using a distributed memory of 2.7 TB. A set of 2.2 million potential markers was derived from these counts using constraints described in the Results section. These constraints were imposed using an extension of the mer-counting software on 960 cores of Edison in 3 minutes of compute time using a distributed memory of 866 GB. The SynOpDH sequences were then individually genotyped against this panel of 2.2 million 50-mer markers using an extension of the mer-counting software running on 1,920 cores of Edison, requiring 23 minutes of compute time. After eliminating two SynOpDH individuals with outlying heterozygosity rates, any remaining markers with heterozygous calls in any individual were screened, leaving 1.7 million high-quality 50 + 1-mer markers. The marker sequences and associated genotype calls are available as Dataset S6 in Additional file 4.

#### Efficient clustering into linkage groups

This marker set was clustered into 21 linkage groups using a novel clustering algorithm (BubbleCluster [41]), which takes advantage of the underlying linear structure of genetic maps to produce a clustering of the markers in just over an hour of run time using one core of a quad-core AMD Opteron 8378 server. For this clustering a LOD threshold of 9 was used, and the resulting clusters included 1.34 million markers with no missing data in at least 46 of the 88 retained individuals. No significant minor clusters were found beyond the largest 21, which ranged in size from 5.2 k to 127.5 k markers.

#### Establishing a framework map

A framework map was derived from the 100,000 markers placed in clusters with the least missing data in the genotype array using MSTmap. This map was found to be in strong agreement (see Results) with the alternative map, which used markers derived from more conventional SNP-finding methods (see above), and is noteworthy in that it is produced directly from analysis of the shotgun sequence, requiring neither an existing assembly nor map (and was generated in less than 3 hours of wall-clock time using software specifically tailored to produce ultra-high-density genetic maps in a high-performance computing environment). Map locations of 50 + 1-mer markers are given in Dataset S7 in Additional file 4.

#### Attaching scaffolds to the map

By the uniqueness property of the underlying k-mers in a meraculous assembly, the set of 50 + 1-mer markers may be directly and uniquely assigned to scaffolds in the assembly by BLAST (or other suitable alignment method) with wordsize 51; 84% of markers in the set are assignable to scaffolds by this technique. These are assigned to 442 k scaffolds spanning 5.28 Gbp (267 k scaffolds larger than 1 kbp spanning 5.23 Gbp). Markers placed in linkage group clusters are assigned to 321 k scaffolds spanning 4.51 Gbp of the assembly (215 k scaffolds larger than 1 kbp spanning 4.48 Gbp). Of scaffolds with two cluster-assigned markers attached, 48/45,805 (0.10%) are found to have markers with conflicting linkage group designations, indicating a very low rate of potential misassembly (or marker mis-assignment). The net separation of marker pairs across this set indicates an inter-chromosomal misassembly rate of no more than one per 3.3 Mbp. We note that this assembly-independent framework map can be extended by identifying k + 1-mer markers on scaffolds, and combining the (sparsely sampled) markers on each scaffold into a haplotype ’super-marker’ with limited missing data. The placement of 50 + 1-mer marker on scaffolds given in Dataset S8 in Additional file 4.

### Nucleotide diversity

We determined the average SNP rate per kilobase between two wheat genotypes by counting all base positions on the concatenated chromosome arm assemblies of cv. Chinese Spring [5] that are polymorphic in the respective pair of accessions and had at least 1× coverage in both W7984 and Opata M85. This analysis was based on the short read alignment against the Chinese Spring assembly (see ‘Read mapping and SNP calling’). Then, we divided this number by the number of all bases of the Chinese Spring assembly that have at least 1× coverage in both W7984 and Opata M85. These calculations were performed separately for the entire genome, the three subgenomes and for coding sequences. The predicted positions of coding sequences on the Chinese Spring assembly [5] (version July 2014) were downloaded from [75]. To find deletions in W7984, we calculated the read depth of the alignments of reads of Opata M85 and W7984 against the assembly of Chinese Spring using the programs ‘samtools depth’ [71] and BEDtools [76].

### Data access

All shotgun reads are deposited into the Short Read Archive, with the following accession numbers: SRP037990, *Triticum aestivum* SynOpDH mapping population; SRP037781, *Triticum aestivum* Synthetic Opata M85; SRP037994, *Triticum aestivum* Synthetic W7984.

The WGS assembly of W7984 is accessible from the European Nucleotide Archive (accession PRJEB7074). The assembly can also be downloaded as a single multi-fasta file from [77]. Digital object identifiers (DOIs) were created with e!DAL [78].

## Additional files

Additional file 1: Figure S1. Distribution of single copy sequences for differing k. **Figure S2.** Estimate of base-level accuracy of W7984 whole genome shotgun assembly. **Figure S3.** Full length cDNA counts versus nucleotide identity. **Figure S4.** Frequency of the Opata M85 allele along the genome. **Figure S5.** Insert size distributions. **Figure S6.** Fraction of cDNA length accounted for by the longest match to a scaffold. **Figure S7.** Number of distinct 51-mers as a function of copy number for pooled SynOpDH reads. **Table S1.** Sequencing summary, *Triticum aestivum* ‘Synthetic W7984’). **Table S2.** Sequencing summary, *Triticum aestivum* ‘Opata M85’. **Table S3.** Shotgun sequencing of SynOpDH individuals. **Table S4.** Summary of W7984 assembly (excluding screened contaminants). **Table S5.** Gap size distributions. **Table S6.** Alignment of *T. aestivum* full length cDNA to assemblies (99% or better nucleotide identity. **Table S7.** Summary statistics of the genetic framework map. Additional file 2:
**Identifiers of full-length cDNAs that can be aligned to the assembly of Chinese Spring with ≥99% identity but not (or with identity <99%) to the assembly of W7984.**
Additional file 3:
**Identifiers of full-length cDNAs that can be aligned to the assembly of W7984 with ≥99% identity but not (or with identity <99%) to the assembly of Chinese Spring.**
Additional file 4:
**Descriptions and Digital Object Identifiers (DOIs) of Datasets S1 to S8.**

## Abbreviations

bp: base pair
DH: doubled haploid
HSP: high-scoring pair
IWGSC: International Wheat Genome Sequencing Consortium
LOD: log-odds
SNP: single nucleotide polymorphism
WGS: whole-genome shotgun
