# Supplementary material for: A whole-genome shotgun approach for assembling and anchoring the hexaploid bread wheat genome
Source: Genome Biol. 2015 Jan 31;16(1):26. doi: 10.1186/s13059-015-0582-8 (PMC4373400; doi:10.1186/s13059-015-0582-8)
Supplement: Additional file 1: Figure S1. — Distribution of single copy sequences for differing k. Figure S2. Estimate of base-level accuracy of W7984 whole genome shotgun assembly. Figure S3. Full length cDNA counts versus nucleotide identity. Figure S4. Frequency of the Opata M85 allele along the genome. Figure S5. Insert size distributions. Figure S6. Fraction of cDNA length accounted for by the longest match to a scaffold. Figure S7. Number of distinct 51-mers as a function of copy number for pooled SynOpDH reads. Table S1. Sequencing summary, Triticum aestivum ‘Synthetic W7984’). Table S2. Sequencing summary, Triticum aestivum ‘Opata M85’. Table S3. Shotgun sequencing of SynOpDH individuals. Table S4. Summary of W7984 assembly (excluding screened contaminants). Table S5. Gap size distributions. Table S6. Alignment of T. aestivum full length cDNA to assemblies (99% or better nucleotide identity. Table S7. Summary statistics of the genetic framework map. [file 13059_2015_582_MOESM1_ESM.docx]

**Supplementary Material**

**Supplementary Figures**

Figure S1. Distribution of single copy sequences for differing k

Figure S2. Estimate of base-level accuracy of W7984 whole genome shotgun assembly

Figure S3. Full length cDNA counts vs. nucleotide identity

Figure S4. Frequency of the Opata M85 allele along the genome

Figure S5. Insert size distributions

Figure S6. Fraction of cDNA length accounted for by the longest match to a scaffold

Figure S7. Number of distinct 51-mers as a function of copy number for pooled SynOpDH reads

**Supplementary Tables**

Table S1. Sequencing summary, *Triticum aestivum* ‘Synthetic W7984’

Table S2. Sequencing summary, *Triticum aestivum* ‘Opata M85’

Table S3. Shotgun sequencing of SynOpDH individuals

Table S4. Summary of W7984 assembly (excluding screened contaminants)

Table S5. Gap size distributions

Table S6. Alignment of *T. aestivum* full length cDNA to assemblies (99% or better nucleotide identity)

Table S7. Summary statistics of the genetic framework map**.**

**Supplementary References**

**Supplementary Figures**


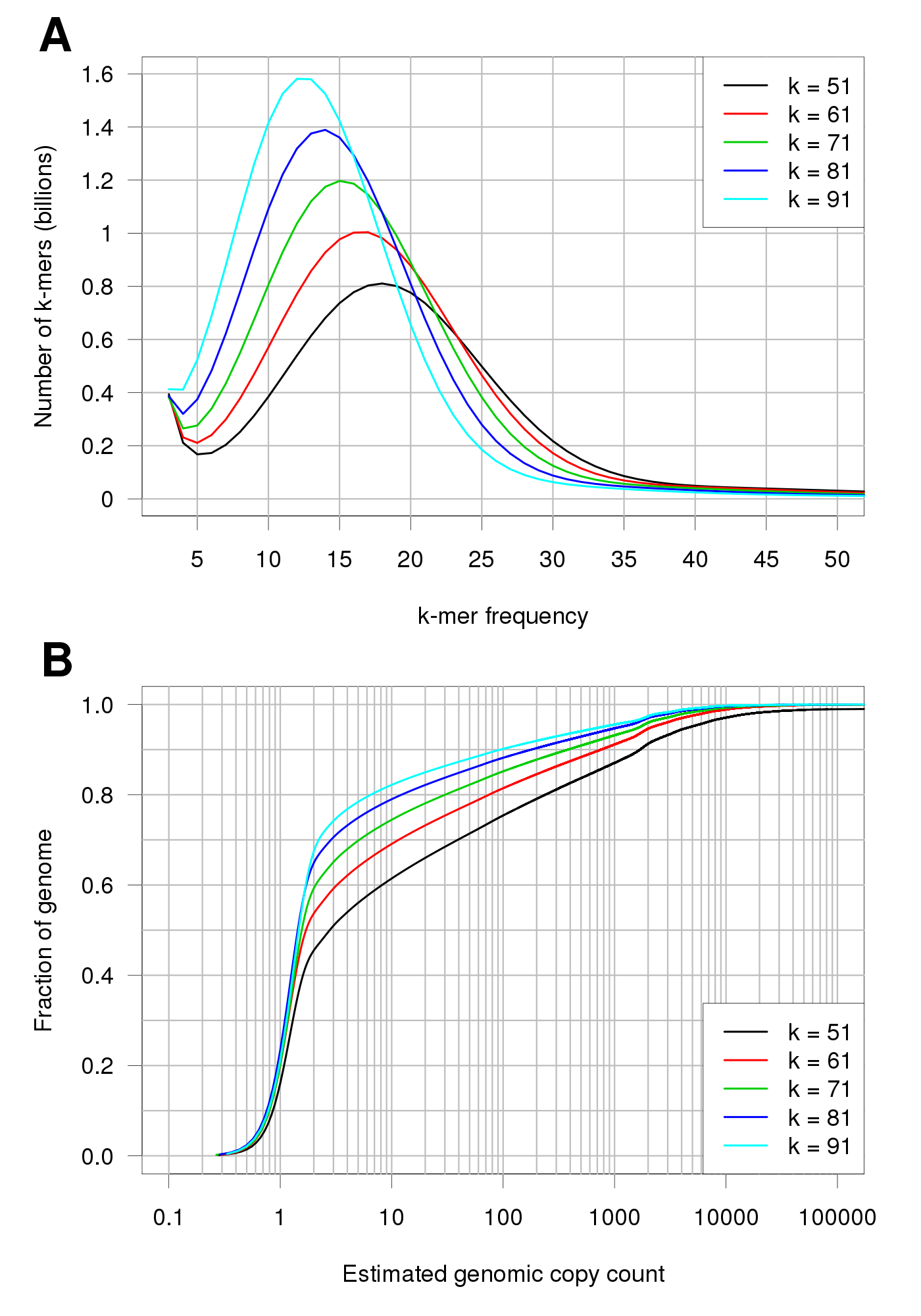


**Figure S1. Distribution of single copy sequences for differing k.** Top panel shows the k-mer frequency distribution for k=51, 61, 71, 81, and 91. Since each read of length R provides (R-k+1) different k-mers, as k increases the k-mer depth decreases, so our W7984 dataset has 51-mer depth ~18-19x but 81-mer depth of only ~14x. Bottom panel shows the cumulative fraction of the wheat genome at varying k-mer copy count. The “knee” at estimated genomic copy count ~1 increases with k, as more of the genome is k-mer unique for larger k than for smaller k. This analysis suggests that ~2/3 of the wheat genome is unique at k = 81, and should be accessible to assembly given sufficient depth.

Discrepancy #1 (a deletion in the reference relative to the assembly):

KC614592.1 GTTCTTGATCAGTGGTATAAGCTTGTATGTTCATGGCGTTCACTGCTC-TATGAACATGATGGCTTTGCAATTGC

Sca1011110 GTTCTTGATCAGTGGTATAAGCTTGTATGTTCATGGCGTTCACTGCTCTTATGAACATGATGGCTTTGCAATTGC

2_0 4166 gttccgttcttgatcagtggtataagcttgtatgttcatggcgttcactgctc-tatgaacatgatggctttgcaattgc

6 88 .....................................................t..........................

4 150 .................................t..........................

5 150 ...........................t..........................

3 150 .........c......t..........................

2 143 ..........................

1 144 ..........................

Discrepancy #2 (A->T substitution):

KC614592.1 TCCCACAAAAATGAGCCAACTTATATAGTATAGCTATTGCGAAAAAATGAATTAGCACCAAA

Sca1011110 TCCCACAAAATTGAGCCAACTTATATAGTATAGCTATTGCGAAAAAATGAATTAGCACCAAA

2_0 1 tcccacaaaaatgagccaacttatatagtatagctattgcgaaaaaatgaattagcacca 60

11 1 ....t................................................. 54

10 1 ........t................................................. 58

9 21 ..........t................................................. 80

8 34 ..........t................................................. 93

7 56 ..........t................................................. 115

6 61 ..........t................................................. 120

5 104 ..........t.................................... 150

4 113 ..........t........................... 150

Discrepancy #3 (C->T substitution):

FJ883565.1 GACTTCAGGAAGATGGAGTACCTCTCACAGGTAACAACATTCCTCCCCATACACTCTGCC

Sca1525215 GACTTCAGGAAGATGGAGTACCTCTCACAGGTAACAACATTCCTCTCCATACACTCTGCC

5_0 61 gacttcaggaagatggagtacctctcacaggtaacaacattcctccccatacactctgcc 120

12 95 .............................................t.............. 36

11 109 .............................................t.............. 50

9 125 .............................................t.............. 66

8 150 .............................................t.............. 91

6 150 ........................................t.............. 96

5 150 ..................t.............. 118

7 150 ..........................................t......t....... 94

10 119 .............................................t.............. 60

Discrepancy #4 (G->C substitution)

FJ883565.1 ACTCTGGTACAGGAGCGTGCACATGGACCCTCAGGTGTACCCTCACCCCAAGAAGTTCGACCCTTC

Scaff1525215 ACTCTGGTACAGGAGCGTGCACATGGACCCTCAGGTGTACCCTCACCCCAACAAGTTCGACCCTTC

5_0 489 ggtgcaactctggtacaggagcgtgcacatggaccctcaggtgtaccctcaccccaagaagttcgacccttc

4 80 .........................................................c..............

2 128 .........................................................c..........

1 150 ................................................c..............

0 150 .........c..............................c..............

**Figure S2. Estimate of base-level accuracy of W7984 whole genome shotgun assembly.** To assess the base-level accuracy of the W7984 shotgun assembly we compared it to six known genic sequences from the W7984 genotype: the three homeologs of the DELLA gene reduced height 1 (Rht-1)[[1](#_ENREF_1)] and of the gibberellin biosynthesis enzyme ent-kaurenoic oxidase (KAO)[[2](#_ENREF_2)]. These six genes provide 15,453 bp of known W7984 sequence; 11,043 bp (71.5%) are covered by scaffolds longer than 1 kbp (78.1% including all scaffolds). Four discrepancies were observed between the W7984 Genbank entries and our shotgun assembly. To determine whether these were errors in our assembly or the Genbank sequence we reviewed the high mapping quality alignments of our W7984 whole genome shotgun reads to these regions. In the following views, the top line is the Genbank entry; the second line is the Scaffold (abbreviated Sca), and the remaining lines are W7984 shotgun reads aligned to the Genbank entry. The discrepancy is shown in red (Genbank entry) and green (meraculous scaffold). For clarity, read sequences are indicated by “.” when they agree with the reference. In each case it is evident that the Genbank entry is not supported by any shotgun reads, while the meraculous assembly is fully supported. Also visible are stray sequencing errors. These results are corroborated by k-mer analysis (data not shown). In each case, the flanking 51-mer to the discrepant base has a unique high quality extension[[3](#_ENREF_3)] to the nucleotide reported in the assembly, and no support for the alternate nucleotide reported in the Genbank entries. We conclude that there are no *bona fide* errors in our assembly over these alignments. It is possible that the entries in Genbank include either errors or variations in the clone of W7984 used in sequencing of the Genbank clones.


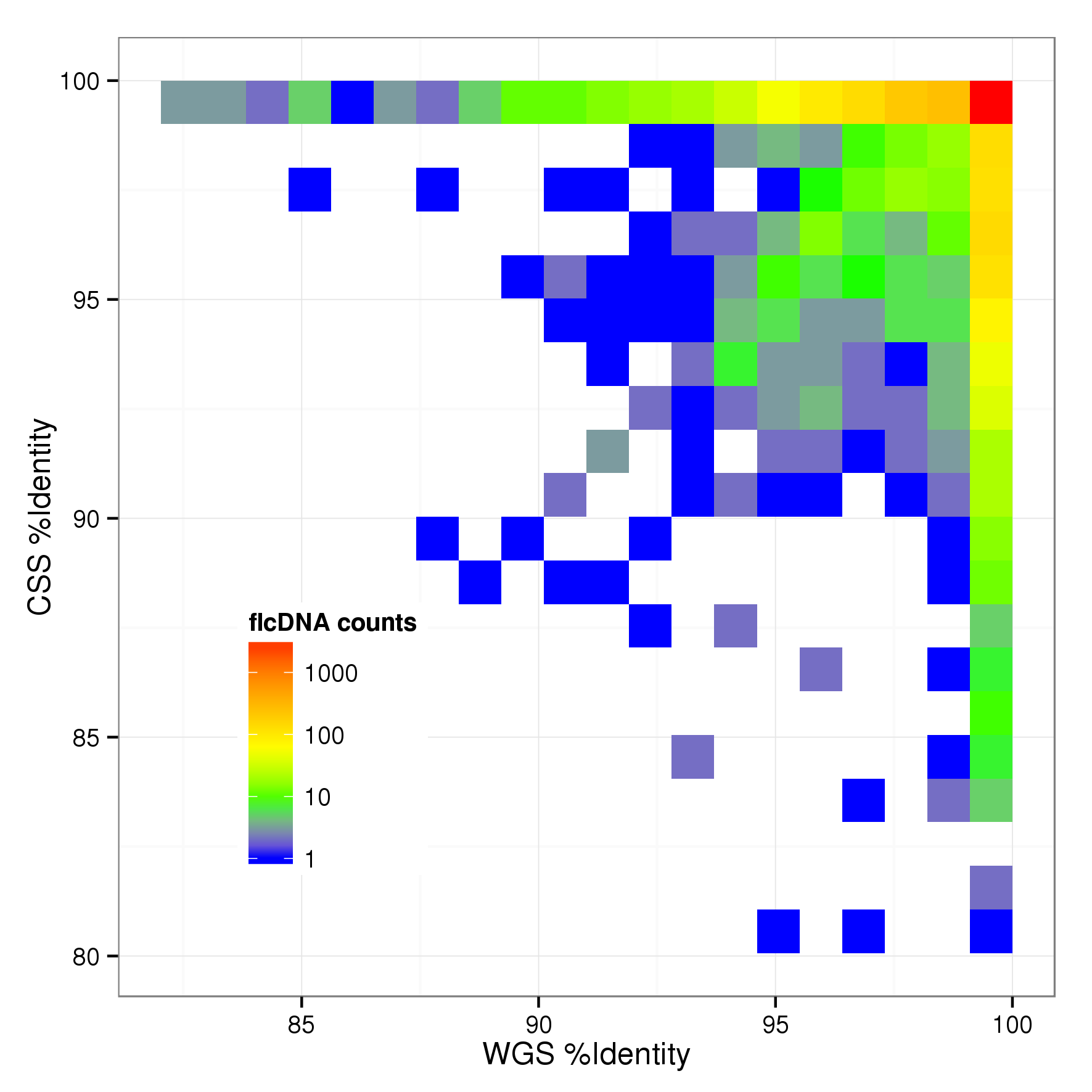


**Figure S3. Full length cDNA counts vs. nucleotide identity.** Heat map shows the number of full length cDNA aligned over >50% of their lengths at the specified percent identity in the Chinese Spring chromosome sorted (CSS) assemblies (vertical axis) and W7984 whole genome shotgun (WGS) assembly. Note that the great majority of counts are in the 99-100% bin for both (upper right hand corner) but there is appreciable density of flcDNA that align at >99% identity vs one assembly but at substantially lower percent identity to the other (stripes along top and right sides).


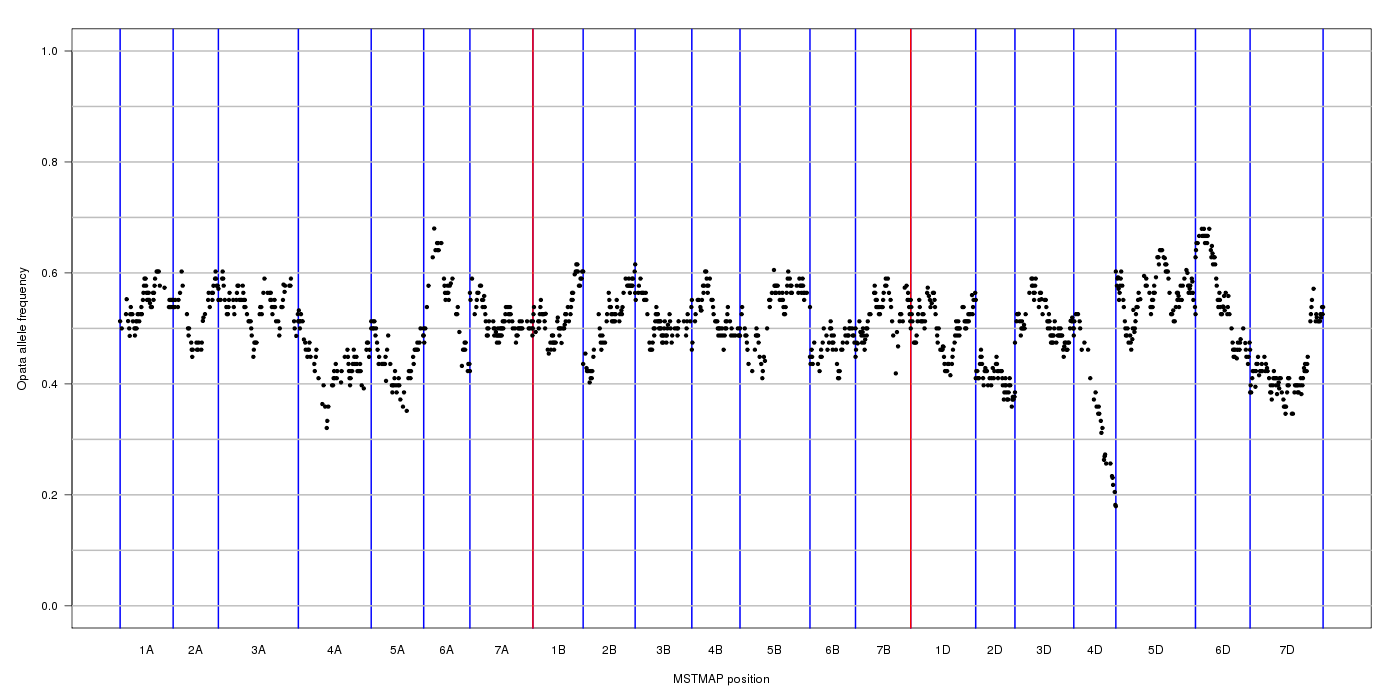


**Figure S4. Frequency of the Opata M85 allele along the genome**. Regions of strong segregation distortion were found on 4D, 6A and 6D.


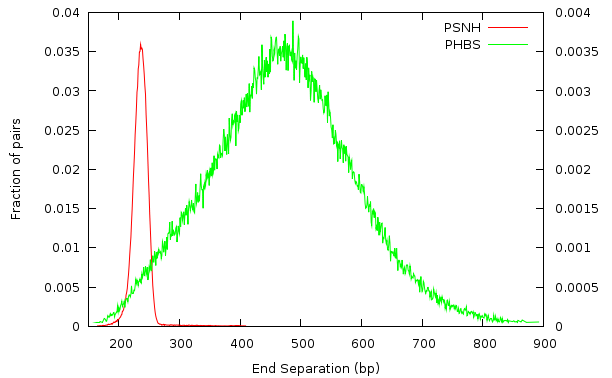


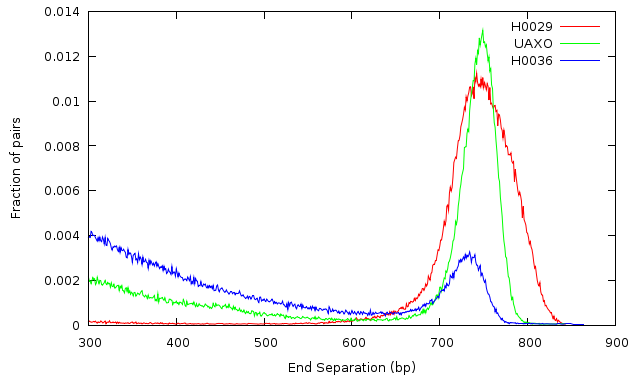


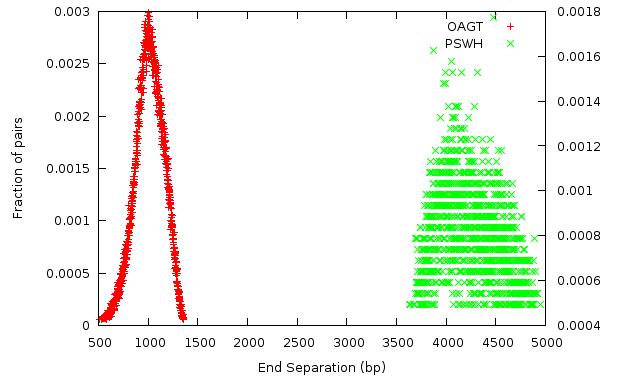


**Figure S5. Insert size distributions.** Top: nominal 250 and 500 bp insert standard fragment libraries. Middle: nominal 800 bp insert fragment libraries. Note that two of the three libraries are bimodal, including substantial weight at short insert sizes. Bottom: mate pair libraries with nominal insert size ~1 kbp and ~4 kbp. Library ID’s are as shown in Table S1.

**Figure S6.** **Fraction of cDNA length accounted for by the longest match to a scaffold**. Only matches with >99% are considered. Each match may include one or more exons aligning to the scaffold. Only one match is shown per cDNA. Red is for whole genome assembly of W7984; blue is for chromosome sorted assembly of ‘Chinese Spring.’ Bars for both assemblies are superimposed. Where they overlap the bar graph appears purple.

**
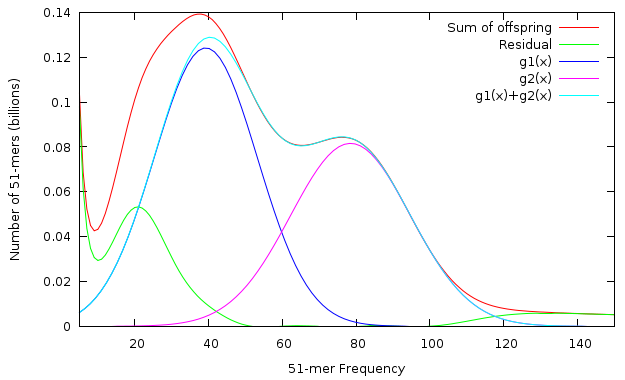
**

**Figure S7. Number of distinct 51-mers as a function of copy number for pooled SynOpDH reads.** Distribution is shown for the 78 progeny used in mapping (excluding 10 progeny with deletions and two contaminated samples). The curves g1(x), g2(x), and g1(x)+g2(x) show fits to double Gaussian distributions, with the mean of g2 (full depth) constrained to be twice of the mean of g1 (half depth=39.3x, corresponding to k-mers that occur in one parental genotype but not the other). Note the residual at low frequency (green) is itself roughly normally distributed, corresponding to ~quarter depth. These are hypothesized to represent heterozygous positions within the nominally homozygous W7984 or Opata lines.

**Supplementary Tables**

**Table S1. Sequencing summary, *Triticum aestivum* ‘Synthetic W7984’**

| Library ID | Mean insert size (standard deviation), bp | Read length, bp | Number of reads (millions) | Total base pairs  (Gbp) | Estimated read depth |
| --- | --- | --- | --- | --- | --- |
| PSNH | 236 +/- 15 | 2x150 | 359 | 53.9 | 3.2x |
| PHBS | 471 +/- 118 | 2x150 | 622 | 93.3 | 5.5x |
| UAXO | bimodal | 2x251 | 346 | 86.7 | 5.1x |
| H0036 | bimodal | 2x251 | 340 | 85.2 | 5.0x |
| H0029 | 735 +/- 75 | 2x251 | 628 | 157.7 | 9.3x |
| OAGT | 1,011 +/- 147 | 2x150 | 355 | 53.2 | 3.1x |
| PSWH | 4,244 +/- 299 | 2x150 | 359 | 53.9 | 3.2x |
| **All short frag** |  |  | 2295 | 476.8 | 28.0x |
| **TOTAL** |  |  | 3009 | 583.9 | 34.3x |

Note: Estimated read depths shown in this table assume genome size of 17 Gbp, which was the genome size previously estimated (IWGSC 2014). This table does not discount low-quality reads, adapter, or other contamination, and estimated read depths are only an approximation.

**Table S2. Sequencing summary, *Triticum aestivum* ‘Opata M85’**

| Library ID | Mean insert size (standard deviation), bp | Read length, bp | Number of reads (millions) | Total base pairs (Gbp) | Estimated read depth |
| --- | --- | --- | --- | --- | --- |
| OBXG | 425 ± 119 | 2x150 | 729 | 109 | 6.4x |
| PHBP | 484 ± 134 | 2x150 | 318 | 47.8 | 2.8x |
| H0030 | 744 ± 53 | 2x251 | 633 | 159 | 9.3x |
| **TOTAL** |  |  |  | 316 | 18.6x |

As in Table S1, “estimated read depth” in Table S5 is given by the total read length divided by 17 Gbp, but does not include any correction for contamination, adapters, or other sequencing artifacts.

**Table S3. Shotgun sequencing of SynOpDH individuals**.

| SynOpDH ID | Index Name | Index (bp) | Well | Est read depth | % artifact | % organelle | Lib name |
| --- | --- | --- | --- | --- | --- | --- | --- |
| 0147 | IT061 | ACCGGC | E8 | 1.6 | 0.2 | 8.5 | NSHZ |
| 0141 | IT058 | ACAAAC | B8 | 1.4 | 0.2 | 10.4 | NSHU |
| 0045 | IT020 | GTGGCC | D3 | 1.3 | 0.3 | 8.0 | NSHS |
| 0142 | IT059 | ACATCT | C8 | 1.4 | 0.2 | 9.7 | NSHX |
| 0035 | IT019 | GTGAAA | C3 | 1.4 | 0.2 | 10.6 | PHOZ |
| 0049 | IT022 | CGTACG | F3 | 1.3 | 0.3 | 12.1 | NSGB |
| 0144 | IT060 | ACCCAG | D8 | 1.6 | 0.1 | 11.4 | NSHW |
| 0048 | IT021 | GTTTCG | E3 | 1.5 | 0.3 | 10.3 | NSGX |
| 0077 | IT030 | CACCGG | F4 | 1.2 | 0.3 | 10.3 | NSGS |
| 0109 | IT043 | TACAGC | C6 | 1.5 | 0.3 | 8.9 | NSHA |
| 0104 | IT042 | TAATCG | B6 | 1.8 | 0.4 | 8.6 | NSHB |
| 0096 | IT038 | CTAGCT | F5 | 1.6 | 0.2 | 11.0 | NSHO |
| 0154 | IT064 | AGAAGA | H8 | 1.1 | 0.4 | 11.7 | NTWA |
| 0137 | IT056 | AAGGAC | H7 | 1.4 | 0.3 | 12.4 | NTWX |
| 0030 | IT015 | ATGTCA | G2 | 1.6 | 0.2 | 9.5 | NTXB |
| 0163 | IT069 | ATACGG | E9 | 0.9 | 0.3 | 8.4 | NTXZ |
| 0172 | IT075 | CCTTAG | C10 | 1.4 | 0.2 | 11.1 | NTYG |
| 0034 | IT018 | GTCCGC | B3 | 1.3 | 0.2 | 9.9 | PHOY |
| 0014 | IT004 | TGACCA | D1 | 1.4 | 0.2 | 9.9 | NSHT |
| 0148 | IT062 | ACGATA | F8 | 1.6 | 0.3 | 10.3 | NSHY |
| 0156 | IT065 | AGATAG | A9 | 1.1 | 0.3 | 11.5 | NTWO |
| 0029 | IT014 | AGTTCC | F2 | 1.6 | 0.1 | 9.4 | NTXO |
| 0026 | IT012 | CTTGTA | D2 | 1.6 | 0.2 | 11.5 | NTXU |
| 0020 | IT009 | GATCAG | A2 | 1.4 | 0.2 | 11.6 | NTXY |
| 0173 | IT076 | CGAGAA | D10 | 1.3 | 0.2 | 10.7 | NTYU |
| 0087 | IT037 | CGGAAT | E5 | 1.2 | 0.2 | 10.6 | PHPY |
| 0138 | IT094 | TTCGAA | F12 | 1.3 | 0.4 | 7.5 | PHPZ |
| 0196 | IT091 | TGCCAT | C12 | 1.1 | 0.1 | 9.8 | NSGG |
| 0074 | IT029 | CAACTA | E4 | 1.3 | 0.2 | 11.0 | NSGP |
| 0111 | IT045 | TCATTC | E6 | 1.4 | 0.2 | 10.6 | NSGY |
| 0097 | IT039 | CTATAC | G5 | 1.3 | 0.4 | 10.0 | NSHP |
| 0125 | IT053 | AACTTG | E7 | 1.3 | 0.6 | 8.3 | NTWW |
| 0120 | IT052 | AACAAA | D7 | 1.3 | 0.2 | 11.2 | NTXP |
| 0168 | IT072 | CCACGC | H9 | 1.5 | 0.4 | 12.0 | NTYO |
| 0033 | IT017 | GTAGAG | A3 | 1.3 | 0.2 | 12.4 | NTYS |
| 0177 | IT079 | GAATAA | G10 | 1.3 | 0.6 | 10.5 | PHOU |
| 0191 | IT087 | GGCACA | G11 | 0.6 | 0.3 | 11.4 | PHPB |
| 0078 | IT031 | CACGAT | G4 | 1.4 | 0.2 | 9.5 | PHPS |
| 0083 | IT033 | CAGGCG | A5 | 1.2 | 0.2 | 10.7 | PHPU |
| 0073 | IT028 | CAAAAG | D4 | 1.1 | 0.3 | 10.9 | NSGO |
| 0112 | IT046 | TCCCGA | F6 | 1.4 | 0.2 | 8.6 | NSGW |
| 0100 | IT040 | CTCAGA | H5 | 1.3 | 0.5 | 10.6 | NSHC |
| 0152 | IT063 | ACTCTC | G8 | 1.3 | 0.4 | 10.8 | NTWB |
| 0159 | IT066 | AGCATC | B9 | 1.3 | 0.2 | 8.9 | NTWG |
| 0117 | IT050 | AAAGCA | B7 | 1.5 | 0.2 | 15.1 | NTXG |
| 0164 | IT070 | ATCCTA | F9 | 1.4 | 0.2 | 9.6 | NTXX |
| 0184 | IT084 | GCACTT | D11 | 1.4 | 0.4 | 11.7 | PHPC |
| 0187 | IT086 | GCTCCA | F11 | 1.4 | 0.2 | 9.8 | PHPH |
| 0086 | IT036 | CCAACA | D5 | 1.5 | 0.3 | 9.0 | PHPW |
| 0193 | IT088 | GGCCTG | H11 | 1.6 | 0.3 | 9.3 | NSGN |
| 0174 | IT095 | TTCTCC | G12 | 1.0 | 0.4 | 7.1 | NSGT |
| 0019 | IT007 | CAGATC | G1 | 1.5 | 0.2 | 10.1 | NTWS |
| 0022 | IT011 | GGCTAC | C2 | 1.3 | 0.2 | 9.8 | NTXS |
| 0165 | IT071 | ATCTAT | G9 | 1.5 | 0.2 | 9.3 | NTXT |
| 0170 | IT073 | CCCATG | A10 | 1.4 | 0.2 | 9.2 | NTYN |
| 0065 | IT024 | GGTAGC | H3 | 1.6 | 0.2 | 12.1 | NTYP |
| 0182 | IT082 | GATGCT | B11 | 1.2 | 0.4 | 9.8 | PHOW |
| 0183 | IT083 | GCAAGG | C11 | 1.3 | 0.8 | 10.6 | PHPG |
| 0185 | IT085 | GCCTTA | E11 | 1.3 | 0.2 | 9.8 | PHPO |
| 0079 | IT032 | CACTCA | H4 | 1.6 | 0.3 | 8.6 | PHPP |
| 0135 | IT093 | TGGCGC | E12 | 1.4 | 0.5 | 9.5 | NSGA |
| 0102 | IT041 | GACGAC | A6 | 1.1 | 0.3 | 9.6 | NSHH |
| 0071 | IT027 | ATTCCT | C4 | 1.5 | 0.3 | 10.7 | NTWH |
| 0140 | IT057 | AATAGG | A8 | 1.1 | 0.3 | 9.9 | NTWT |
| 0132 | IT055 | AAGCGA | G7 | 1.2 | 0.9 | 10.8 | NTWY |
| 0069 | IT026 | ATGAGC | B4 | 1.4 | 0.3 | 7.9 | NTXA |
| 0031 | IT016 | CCGTCC | H2 | 1.5 | 0.1 | 10.3 | NTXC |
| 0028 | IT013 | AGTCAA | E2 | 1.5 | 0.2 | 9.3 | NTXH |
| 0118 | IT051 | AAATGC | C7 | 1.3 | 0.2 | 11.9 | NTXN |
| 0115 | IT048 | TCGGCA | H6 | 1.3 | 0.5 | 13.0 | NTYA |
| 0162 | IT068 | AGGCCG | D9 | 1.0 | 0.3 | 10.4 | NTYC |
| 0175 | IT077 | CTGCTG | E10 | 1.5 | 0.2 | 10.2 | NTYT |
| 0085 | IT035 | CATTTT | C5 | 1.2 | 0.3 | 11.1 | PHPX |
| 0110 | IT044 | TATAAT | D6 | 1.4 | 0.2 | 9.1 | NSGZ |
| 0010 | IT003 | TTAGGC | C1 | 1.6 | 0.2 | 11.4 | NSHN |
| 0015 | IT005 | ACAGTG | E1 | 1.9 | 0.2 | 7.8 | NTWC |
| 0021 | IT010 | TAGCTT | B2 | 1.5 | 0.3 | 9.4 | NTYB |
| 0171 | IT074 | CCGCAA | B10 | 1.5 | 0.2 | 10.4 | NTYH |
| 0051 | IT023 | GAGTGG | G3 | 1.6 | 0.1 | 8.6 | PHPN |
| 0084 | IT034 | CATGGC | B5 | 1.3 | 0.3 | 10.8 | PHPT |
| 0199 | IT092 | TGCTGG | D12 | 1.4 | 0.2 | 8.7 | NSGC |
| 0194 | IT090 | TGAATG | B12 | 1.3 | 0.2 | 8.2 | NSGH |
| 0114 | IT047 | TCGAAG | G6 | 0.9 | 0.4 | 12.1 | NSGU |
| 0005 | IT002 | CGATGT | B1 | 1.5 | 0.2 | 9.3 | NSHG |
| 0018 | IT006 | GCCAAT | F1 | 1.6 | 0.1 | 9.8 | NTWN |
| 0161 | IT067 | AGCGCT | C9 | 1.4 | 0.2 | 10.5 | NTWP |
| 0116 | IT049 | AAACAT | A7 | 1.0 | 0.3 | 10.4 | NTWZ |
| 0067 | IT025 | ACTGAT | A4 | 1.4 | 0.4 | 10.2 | NTXW |
| 0179 | IT080 | GACGGA | H10 | 1.2 | 0.5 | 10.2 | NTYW |
| 0181 | IT081 | GATATA | A11 | 1.3 | 0.5 | 10.8 | PHOX |

**Table S4. Summary of W7984 assembly (excluding screened contaminants)**

| Min. scaffold length (bp) | Number of scaffolds | Number of contigs | Total scaffold length | Total contig length | % contig |
| --- | --- | --- | --- | --- | --- |
| All | 6,870,110 | 8,141,183 | 9.134 Gbp | 7.883 Gbp | 86.30% |
| 100 | 6,870,110 | 8,141,183 | 9.134 Gbp | 7.883 Gbp | 86.30% |
| 250 | 1,565,460 | 2,826,042 | 8.429 Gbp | 7.178 Gbp | 85.15% |
| 500 | 955,122 | 2,167,349 | 8.214 Gbp | 6.965 Gbp | 84.79% |
| 1k | 645,811 | 1,803,494 | 8.001 Gbp | 6.763 Gbp | 84.52% |
| 2.5k | 446,901 | 1,532,809 | 7.700 Gbp | 6.479 Gbp | 84.13% |
| 5k | 377,447 | 1,399,647 | 7.439 Gbp | 6.262 Gbp | 84.17% |
| 10k | 253,986 | 1,100,602 | 6.554 Gbp | 5.581 Gbp | 85.15% |
| 25k | 94,482 | 558,357 | 3.975 Gbp | 3.441 Gbp | 86.55% |
| 50k | 21,110 | 178,460 | 1.460 Gbp | 1.278 Gbp | 87.58% |
| 100k | 1,611 | 20,760 | 198.5 Mbp | 176.1 Mbp | 88.73% |
| 250k | 2 | 54 | 522.7 kbp | 456.7 kbp | 87.36% |

**Table S5. Gap size distributions**

| Gap size range (bp) | Number of gaps | Total length of gaps in this range (Mbp) | Gaps of this size or shorter | % total gaps of this size or shorter | Total length of gaps this size or shorter (Mbp) | % total gap length this size or shorter |
| --- | --- | --- | --- | --- | --- | --- |
| 1 - 99 | 309,404 | 8.8 | 309,404 | 24.34% | 8.8 | 0.70% |
| 100 - 199 | 76,654 | 11.0 | 386,058 | 30.37% | 19.8 | 1.58% |
| 200 - 299 | 64,360 | 16.1 | 450,418 | 35.44% | 35.9 | 2.87% |
| 300 - 399 | 68,471 | 24.0 | 518,889 | 40.82% | 59.8 | 4.78% |
| 400 - 499 | 68,606 | 30.8 | 587,495 | 46.22% | 90.6 | 7.24% |
| 500 - 599 | 58,875 | 32.3 | 646,370 | 50.85% | 122.9 | 9.82% |
| 600 - 699 | 47,690 | 30.9 | 694,060 | 54.60% | 153.8 | 12.29% |
| 700 - 799 | 41,787 | 31.2 | 735,847 | 57.89% | 185.0 | 14.78% |
| 800 - 899 | 33,764 | 28.6 | 769,611 | 60.55% | 213.6 | 17.07% |
| 900 - 999 | 30,931 | 29.4 | 800,542 | 62.98% | 243.0 | 19.41% |
| 1k - 2.5k | 314,952 | 524.2 | 1,115,494 | 87.76% | 767.1 | 61.30% |
| 2.5k - 5k | 155,495 | 483.9 | 1,270,989 | 99.99% | 1,251.1 | 99.97% |
| 5k - 9k | 84 | 0.423 | 1,271,073 | 100.00% | 1,251.5 | 100.00% |

**Table S6. Alignment of *T. aestivum* full length cDNA to assemblies (99% or better nucleotide identity).**

|  | This report/W7984 | IWGSC/Chinese Spring |
| --- | --- | --- |
| Transcripts aligning (>50% length) | 4,663/6,000 (77.7%) | 4,580/6,000 (76.3%) |
| % length in single best hit (of transcripts aligning) | 8.91 MB/9.92 MB (89.8%) | 8.25 MB/9.69 MB (85.2%) |
| Aligned seq (any length) | 5,747/6,000 (95.8%) | 5,863/6,000 (97.7%) |
| Aligned seq (at least 25%) | 5,135/6,000 (85.6%) | 5,362/6,000 (89.4%) |
| Aligned seq (at least 50%) | 4,663/6,000 (77.7%) | 4,580/6,000 (76.3%) |
| Aligned seq (at least 75%) | 4,090/6,000 (68.2%) | 3,590/6,000 (59.8%) |

**Table S7. Summary statistics of the genetic framework map.**

| chromosome | length in cM | Number of bins | Average distance between neighboring markers (cM) | Maximum distance between neighboring markers (cM) |
| --- | --- | --- | --- | --- |
| 1A | 117.878 | 56 | 2.1 | 9.2 |
| 2A | 92.517 | 43 | 2.2 | 6.9 |
| 3A | 172.2 | 77 | 2.3 | 9.2 |
| 4A | 156.679 | 71 | 2.2 | 6.9 |
| 5A | 111.967 | 59 | 1.9 | 5.8 |
| 6A | 99.391 | 42 | 2.4 | 9.2 |
| 7A | 135.625 | 71 | 1.9 | 6.9 |
| 1B | 113.814 | 60 | 1.9 | 4.6 |
| 2B | 111.506 | 60 | 1.9 | 6.9 |
| 3B | 122.478 | 62 | 2 | 10.4 |
| 4B | 102.696 | 50 | 2.1 | 5.7 |
| 5B | 155.004 | 75 | 2.1 | 9.2 |
| 6B | 97.872 | 52 | 1.9 | 8 |
| 7B | 118.551 | 66 | 1.8 | 5.7 |
| 1D | 136.487 | 81 | 1.7 | 4.6 |
| 2D | 85.027 | 55 | 1.6 | 3.4 |
| 3D | 126.448 | 71 | 1.8 | 8 |
| 4D | 90.119 | 30 | 3.1 | 9.2 |
| 5D | 170.702 | 95 | 1.8 | 8 |
| 6D | 121.074 | 65 | 1.9 | 6.9 |
| 7D | 157.445 | 94 | 1.7 | 4.6 |
| sum | 2826.48 | 1335 |  |  |

**Supplemental References**

1. Wilhelm EP, Mackay IJ, Saville RJ, Korolev AV, Balfourier F, Greenland AJ, Boulton MI, Powell W: **Haplotype dictionary for the Rht-1 loci in wheat.** *Theoretical and Applied Genetics* 2013, **126:**1733-1747.

2. Khlestkina EK, Kumar U, Röder MS: **Ent-kaurenoic acid oxidase genes in wheat.** *Molecular Breeding* 2010, **25:**251-258.

3. Chapman JA, Ho I, Sunkara S, Luo S, Schroth GP, Rokhsar DS: **Meraculous: de novo genome assembly with short paired-end reads.** *PLoS ONE* 2011, **6:**e23501.
