# Supplementary material for: A whole-genome shotgun approach for assembling and anchoring the hexaploid bread wheat genome
Source: Genome Biol. 2015 Jan 31;16(1):26. doi: 10.1186/s13059-015-0582-8 (PMC4373400; doi:10.1186/s13059-015-0582-8)
Supplement: Additional file 4: — Descriptions and Digital Object Identifiers (DOIs) of Datasets S1 to S8. [file 13059_2015_582_MOESM4_ESM.docx]

**Dataset S1**. Deletions (≥50 bp) in W7984 relative to Chinese Spring and Opata M85. Genetic positions of contigs harboring the deleted intervals are included if available. Both the start and the end coordinates are 1-based. DOI: **dx.doi.org/10.5447/IPK/2014/17**

Dataset S2: SNP positions on the WGS assembly of Synthetic W7984 and associated genotype calls in the mapping population. Genotypes are encoded as 0 (Synthetic W7984), 2 (Opata M85) and NA (missing data). DOI: **dx.doi.org/10.5447/IPK/2014/8**

Dataset S3: Genetic positions and genotype scores of framework markers. Framework markers are consensus genotypes that were constructed by aggregating SNP information on the scaffold level. Genotype scores are given as a character string with the following encoding: 0 (Opata M85), 2 (Synthetic W7894), 3 (missing data). The order of individuals is given in the first comment line of the file. Note that the encoding for the two parents has been switched relative to Dataset S2. Summary statistics for the genetic map are given in Table S7. DOI: **dx.doi.org/10.5447/IPK/2014/9**

Dataset S4: Genetic positions and consensus genotypes of 437,973 scaffolds. Genotype scores are encoded as in Dataset S3. DOI: **dx.doi.org/10.5447/IPK/2014/10**

Dataset S5: Genetic positions of 1,552,095 contigs of the sorted chromosome arm assemblies of cv. Chinese Spring. DOI: **dx.doi.org/10.5447/IPK/2014/16**

Dataset S6: Genotypes for 1,716,090 50 + 1-mer markers across 88 individuals defined as described in the results (Ultradense Genetic Linkage Map) and Methods sections (K-mer based genetic map). Genotypes are encoded as 1 (Synthetic W7984), −1 (Opata M85), or 0 (missing data). Markers are labeled by their defining 50-mer sequence tag. DOI: **dx.doi.org/10.5447/IPK/2014/11**

**Dataset S7**: Map locations for 1,658,636 50 + 1-mer markers successfully placed in one of the 21 major linkage groups. Markers are labeled by their defining 50-mer sequence tag and the cluster identity and map location found as described in Method section (K-mer based genetic map). DOI: **dx.doi.org/10.5447/IPK/2014/12**

Dataset S8: Placement of 50 + 1-mer markers on scaffolds by 51-mer match for 1,856,299 markers. As described in the Methods section, markers are defined by their shared 50-mer prefix found in both parental strains and a 51st base unique to each parent. As such, placement of markers on W7984 scaffolds is determined by unique 51-mer match. Match details are described in the header to the file. DOI: **dx.doi.org/10.5447/IPK/2014/13**
